# Activated SOX9+ renal epithelial cells promote kidney repair through secreting factors

**DOI:** 10.1111/cpr.13394

**Published:** 2023-01-04

**Authors:** Hao Nie, Zixian Zhao, Dewei Zhou, Dandan Li, Yujia Wang, Yu Ma, Xutao Liu, Wei Zuo

**Affiliations:** ^1^ East Hospital, School of Medicine Tongji University Shanghai China; ^2^ Super Organ R&D Center Regend Therapeutics Shanghai China; ^3^ Samueli School of Engineering University of California Los Angeles Los Angeles California USA; ^4^ Key Laboratory of Transplant Engineering and Immunology, Ministry of Health, West China Hospital Sichuan University Chengdu China

## Abstract

A broad spectrum of lethal kidney diseases involves the irreversible destruction of the tubular structures, leading to renal function loss. Following injury, a spectrum of tissue‐resident epithelial stem/progenitor cells are known to be activated and then differentiate into mature renal cells to replace the damaged renal epithelium. Here, however, we reported an alternative way that tissue‐resident cells could be activated to secrete multiple factors to promote organ repair. At single‐cell resolution, we showed that the resident SOX9+ renal epithelial cells (RECs) could expand in the acutely injured kidney of both mouse and human. Compared to other cells, the SOX9+ RECs overexpressed much more secretion related genes, whose functions were linked to kidney repair pathways. We also obtained long‐term, feeder‐free cultured SOX9+ RECs from human urine and analysed their secretory profile at both transcriptional and proteomic levels. Engraftment of cultured human SOX9+ RECs or injection of its conditional medium facilitated the regeneration of renal tubular and glomerular epithelium, probably through stimulating endogenous REC self‐activation and mediating crosstalk with other renal cells. We also identified S100A9 as one of the key factors in the SOX9+ REC secretome. Altogether, the abilities to extensively propagate SOX9+ RECs in culture whilst concomitantly maintaining their intrinsic secretory capacity suggest their future application in cell‐free therapies and regeneration medicine.

## Graphical abstract

The secretome of SOX9+ RECs, especially soluble S100A9 protein, could play a critical role in renal tubules regeneration by triggering resident SOX9+ REC proliferation and intercellular communications among adjacent tubular epithelial cells (TECs), immune cells and vascular endothelial cells (VECs).

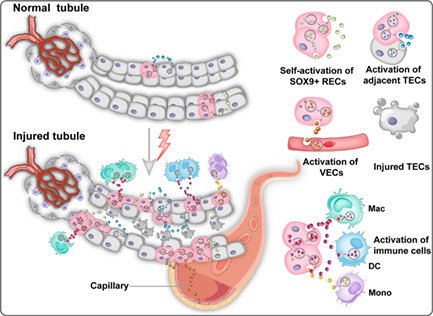

## 1 INTRODUCTION

Kidney diseases affect about one in every 10 people and have high morbidity and mortality.^1^ Acute kidney injury (AKI) is a significant kidney disease featured by rapid decline and even loss of renal function.^2^ The leading causes of AKI include sepsis, nephrotoxins, renal ischemia–reperfusion injury, so on,^3^ only a few treatments exist.^4^ Fortunately, the kidneys can undergo facultative regeneration because of unique cell types, which are the major contributors to maintaining renal homeostasis and tubular restoration.^5^, ^6^ Thus, cell‐based strategies have the biggest potential to overcome clinical therapy challenges.^7^

Sex‐determining region Y box (SOX) is a family of transcription factors, which is previously known as mediators of fetal renal development. However, controversy remains concerning the identity of SOX9+ cells responsible for mature epithelial repair after kidney injury.^8^ Researchers noticed that a rare population of SOX9 positive progenitor cells was represented in the proximal tubule of healthy adult kidneys,^4^, ^9^, ^10^ whereas widely stimulated in calbindin‐28dk+ distal convoluted tubules^9^ and parietal epithelial cells after injury.^11^ Coupling genetic lineage tracing studies revealed the identity of activated SOX9+ populations as the critical intrinsic molecular driver of early injury response.^12^, ^13^, ^14^, ^15^, ^16^, ^17^, ^18^ For example, Kumar et al. indicated that over 40% of SOX9+ cells re‐enter mitosis after ischemic injury, contributing to regenerating functional proximal tubule epithelium.^7^ Besides, the findings from Kang et al.^16^ and our lab^10^ further proved that the descendants of SOX9 positive cells contributed to multiple segments of epithelial repair, including proximal tubule, Henle's loop, distal tubule, collecting duct and the parietal layer of glomerulus. In summary, original researchers thought SOX9+ RECs might be the core of epithelial restoration by dedifferentiation and re‐differentiation.^19^ However, the exact repair mechanism remains unclear.

Recently, a body of evidence has bolstered that the paracrine mechanism is also responsible for AKI therapy.^8^, ^20^, ^21^ The secretome is defined as the complex array of soluble molecules and extracellular vesicles (EVs) that create a microenvironment for cellular renewal.^8^, ^22^ These bioactive factors have the therapeutic functions of anti‐fibrotic,^23^ angiogenic, antioxidative, mitogenic and anti‐apoptotic.^24^, ^25^ Previous studies mainly focused on the secretome of mesenchymal stem cells (MSCs). For example, Tögel and colleagues demonstrated that intra‐carotid administration of MSCs observably accelerated acute kidney damage recovery 48 h after ischemia, even though no transplanted cells resided in kidney tubules after 24 h.^8^, ^26^ The results were congruent with another studying showing that MSC‐derived conditional medium (CM) attenuated renal tubular cell apoptosis in a cisplatin‐induced kidney injury model. In those experiments, the authors indicated that MSC‐derived secreted factors enhanced the migration and proliferation of the proximal tubular epithelium *in vivo* and *in vitro*.^27^ Besides MSC, adult kidney‐derived CD133+ cells were also shown to ameliorate tubular dilation and fibrosis by intravenous injection without being mediated by self‐homing to the kidneys.^28^ These findings support the idea that the secretome administration plays a critical role in renal protection and repair.

In our study, we identified the endogenous SOX9+ RECs could be activated after damage in mouse and human. Single‐cell transcriptional analysis revealed the secreted signature of SOX9+ RECs, whose function contributed to renal restoration. Based on a feeder‐free culture system, we showed that the long‐term, cultured SOX9+ RECs could participate in renal tubular and glomerular epithelium regeneration by secretory function.

## 2 MATERIALS AND METHODS

### 2.1 Mouse kidney injury models

All animal experiments were carried out in accordance with Chinese National Guidelines GB/T 35892–20181, as well as under the guidance of the Institutional Animal Care and Use Committee of Tongji University. To establish a unilateral partial nephrectomy (UPN) mouse model, 8–10 weeks C57BL/6 mice were euthanized by intraperitoneal injection of 3.7% chloral hydrate (0.5 g/kg body weight). The left lateral peritoneum was cut to expose the left kidney, and the renal artery was clamped with hemostatic forceps. About one‐third of the left kidney was cut off from the upper pole of the kidney using a surgical blade. After cleaning off the blood, the incision was sealed with FuAiLe Medical glue (FAL), and the hemostatic forceps on the renal artery were removed. The muscle layer was closed with sutures (Ethicon, Germany), followed by the closing of the skin. For the unilateral ureteral obstruction (UUO) injury model, the abdomen was opened with a midline incision and the left kidney and upper ureter were exposed. Mice were subjected to surgical cautery of the left ureter 15 mm below the pelvis. Kidney samples were harvested on day 0, day 1, day 10 and day 20 post‐surgery for analysis. Sham surgery kidneys in UPN and UUO models were also harvested.

For the unilateral ischemia–reperfusion injury (UIRI) model, 6–8 weeks NOD SCID mice were anaesthetised using 3.7% chloral hydrate (0.5 g/kg body weight), and the mice were put prostrate on a heating pad at a temperature of 37°C for the duration of the surgical procedure. The left renal pedicle was occluded for 30–40 min using a microaneurysm clamp, during which time the kidney was moistened by phosphate‐buffered saline (PBS) every 3 min. Then the clamp was removed, and reperfusion was confirmed by observing tissue colour change. The kidney was returned to the abdomen with an intraperitoneal injection of 200 μl penicillin/streptomycin (P/S) to prevent infection. For sham operation, mice had only incisions in the skin and muscle layer, but the renal pedicles were not clamped.

For the Adriamycin (ADR) induced glomeruli injury model, BALB/C mice of 8 weeks were treated with a single dose of ADR (Sangon Biotech), 10.5 mg/kg, by tail vein injection. All mouse injury experiments were performed on male mice and were randomly allocated to the experimental groups.

### 2.2 Mouse and human renal tissues

For mouse REC cloning, 8–10 weeks mice bred in a specific pathogen free (SPF) facility were collected to obtain renal cortex, medulla and papilla samples. For human kidney tissue sampling, percutaneous renal needle biopsies were performed to obtain patient tissue with membranous nephropathy (MN) by ultrasound‐guided core tissue biopsy needles (18 gauge). All renal specimens were subjected to pathological diagnosis. All the human renal tissues were obtained following clinical standard operating procedure (SOP) under the patient's consent and approved by the Hospital Ethics Committee.

### 2.3 SOX9+ RECs isolation and expansion

For RECs isolation from kidney tissues, samples were washed with cold wash buffer (F12 medium containing 5% fetal bovine serum [FBS] and 1% P/S) and minced by sterile scalpel into 0.2–0.5 mm^3^ sizes to a viscous and homogeneous appearance. The minced tissue was then digested with dissociation buffer including DMEM/F12 (Gibco, USA), 2 mg/ml protease XIV (Sigma, USA), 0.01% trypsin (Gibco, USA) and 10 ng/ml DNase I (Sigma, USA) in 37°C incubator 2 h with gentle agitation. Digested cell suspensions were washed with cold‐wash buffer and passed through 70 μm Nylon mesh (Falcon, USA) to remove aggregates. Cell pellets were collected by centrifuge of 200*g* and then seeded onto a feeder layer of lethally irradiated 3T3‐J2 cells in modified SCM‐6F8 medium. Human SOX9+ RECs were generated from urine samples and expanded as previously described.^29^

For better visualization of colony growth, td‐Tomato+ RECs derived from mT/mG mice were used. For GFP labelling of cultured SOX9+ RECs, medium containing lentivirus was added to the cell culture together with 10 μg/ml polybrene (1:2000)^29^ and cell identities were analysed by CytoFLEX LX flow cytometry with appropriate markers before use (SOX9+, ATP1A1− and CDH1−). Analysis was gated using forward and side scatters characteristics and corresponding positive/negative control. Data were analysed using the flow cytometry software, FlowJo (TreeStar).

### 2.4 Quantitative real time‐PCR (RT‐qPCR)

Total RNA was prepared from cultured RECs using a Rneasy Mini kit (QIAGEN) according to the manufacturer's instructions. All RNA was digested with DNase I (Takara, Japan). One micro gram total RNA and PrimeScript RT Master Mix (Takara, Japan) was used for reverse transcription in a SimpliAmp Thermal Cycler (Life Technologies, USA). RT‐qPCR was performed in triplicate using a QuantStudio3 Sequence Detection System and SYBR Premix Ex Taq II (Takara, Japan). DNA primer pairs were designed to span exons, when possible, to ensure that the product was from mRNA. The following cycling conditions were used: 1 cycle of 95°C for 30 s, 35 cycles of 95°C for 5 s and 60°C for 34 s. The specificity of the amplified product was evaluated using the melting curve analysis. Internal control glyceraldehyde 3‐phosphate dehydrogenase (GAPDH) was used to normalize the result in each reaction, and relative fold change was calculated by the 2^−ΔΔCt^ method. The following primer pairs were used:

**Table:** 

	Gene name	Forward primer	Reverse primer
Human	GAPDH	AGTATGACAACAGCCTCAAGAT	GTCCTTCCACGATACCAAA
	S100A9	ATGCTGATGGCGAGGCTAAC	CCACTGTGGTCTTAGGGGGT
Mouse	GAPDH	CGGAGTCAACGGATTTGGTCGTAT	AGCCTTCTCCATGGTGGTGAAGAC
	Sox9	AGCACAAGAAAGACCACCCC	ATGTGAGTCTGTTCCGTGGC
	Pax2	GGGAAGCTACCCTACCTCCA	TGCTGAATCTCCAAGCCTCA

### 2.5 Histology and immunofluorescence

For cells immunofluorescent (IF) staining, cells were fixed by 3.7% formaldehyde and then permeabilized with 0.2% Triton X‐100 for 5–8 min. For cryo‐section, tissue samples were fixed in 3.7% formalin overnight at 4°C, then embedded by the Tissue‐Tek O.C.T compound (Sakura, Japan) after PBS washed, frozen sections (5‐μm thick) were made by cryotome (Leica microsystem, Germany). For the paraffin section, the fixed tissue was dehydrated by ethanol gradient processed in an automatic tissue processor and then embedded into the paraffin blocks. Five to seven 5–7 micrometre sections were using a microtome (Leica microsystem, Germany). Fixed and paraffin sections were further blocked with 10% donkey serum. Then followed by incubation with indicated primary antibody overnight at 4°C, and incubation with secondary antibody for 2 h. Fluorescent images were visualized and captured with an Olympus IX73 microscope. Quantitative image analyses were performed using the ImageJ software. Haematoxylin and eosin (H&E) staining was performed in standard protocol. The tubular injury score was determined as followed: 0, 0%–5%; 1, 5%–10%; 2, 11%–25%; 3, 26%–45%; 4, 46%–75% and 5, >76%.^30^

Antibodies used for immunofluorescence include:

**Table:** 

Antibodies	Brand	Catalogue	Working dilution
SOX9	Abways	CY5400	1:200
Ki67	BD Pharmingen	550609	1:100
PAX2	R&D	AF3364	1:50
ATP1A1	Santa Cruze	C464.6	1:200
AQP1	Abways	CY6767	1:200
GFP	Abcam	ab6673	1:500
UMOD	Santa Cruze	Sc‐271022	1:100
SLC12A1	Proteintech	18970‐1‐AP	1:100
CDH1	Abcam	Ab40772	1:100
KIM1	R&D	AF1817	1:100
SYNPO	Proteintech	21064‐1‐AP	1:100
FN1	Abcam	ab45688	1:200
S100A9		ab9250	
Alexa Fluor‐conjugated Donkey 488/594	Life Technologies		1:200

### 2.6 Western blotting

Cells were digested and lysed in RIPA buffer (CST) containing protease inhibitors cocktail (Roche) followed by standard Western blotting procedure.^31^ To detect the 13‐kD S100A9 expression, SOX9+ RECs derived CM were centrifuged through Ultra‐15 10K Centrifugal Filter Device (Amicon, Millipore) at 4000*g* for 45 min at 4°C to collect concentrate whose mass was more 10‐kD and then overnight freeze‐drying. After measuring protein concentration, samples were loaded and separated on 15% precast polyacrylamide gels, and then transferred to PVDF membranes (Roche) at 300 mA for 30 min. Membranes were blocked with 5% no‐fat powdered milk, and then incubated with primary antibodies overnight, followed by secondary antibodies. The specific signals were detected by Immobilon Western Chemiluminescent HRP Substrate (Millipore) and Tanon image system.

Antibodies used for Western blotting include: S100A9 (Abcam, ab92507), α‐tubulin (Abways Technology, ab0048) and HRP‐conjugated anti‐rabbit IgG (H + L) as a secondary antibody (Beyotime, A0208).

### 2.7 Urine sample collection and processing

All steps were performed on ice or at 4°C. Urine samples were collected from 52 (12 + 40) healthy volunteers with two different age ranges. Urine specimens from 40 healthy older volunteers were obtained from the department of nephrology in Tongji Hospital, Tongji University. Midstream of voided urine specimen was collected for each donor in the morning. Once the urine volume was measured, the sample was transferred into a 50 ml conical tube and centrifuged immediately at the speed of 390*g* and the cell pellets were washed twice with cold wash buffer including F12 medium, 5% FBS (Hyclone, Australia), 1%P/S (Life, 15070‐063), 1% l‐glutamine (Life, 25030‐081) and resuspended in cold PBS. Details were prepared as described.^29^ Sorted cells were subjected to single‐cell RNA sequencing (sc‐RNA‐seq) immediately. Due to the limited cell number in the urine samples, the samples were combined based on groups (Younger urine, Older urine).

### 2.8 Urinary single‐cell clustering analysis

Single cells were captured and barcoded in 10× Chromium Controller (10× Genomics). Subsequently, RNA from the barcoded cells was reverse‐transcribed, and sequencing libraries were prepared using Chromium Single Cell 3'v3 Reagent Kit (10× Genomics) according to the manufacturer's instructions. Sequencing libraries were loaded on an Illumina NovaSeq with 2 × 150 paired‐end kits at Novogene, China. Raw sequencing reads were processed using the Cell Ranger v.3.1.0 pipeline from 10X Genomics. Data were aggregated and normalized to the same sequencing depth, resulting in a combined gene‐barcode matrix of all samples. Seurat R package (version 4.0.2) was used for dataset analysis. According to the number of detected genes and the percentage of mitochondrial genes in the urine samples, low‐quality cells with mitochondrial gene content >25% and detected genes less than 200 or more than 9000 were removed. This filtering step resulted in 15,760 genes × 1010 cells sampled from the younger urine species, and 8594 genes × 1774 cells from the older urine species. Then the function *NormalizeData* in Seurat was used to scale and log transform the dataset. Two thousand highly variable genes were identified by using the *FindVariableGenes* function. *ScaleData* function regressed out the variants arising from library size and percentage of mitochondrial genes. Principal component analysis (PCA) was performed on the variable genes with *RunPCA* function. Additionally, uniform manifold approximation and projection (UMAP) was used on the top 50 principal components for visualizing the cells. *FindClusters* function was used to identify cell clusters with a resolution of 0.2, which produced seven clusters. *VlnPlot* function showed the markers used to define each cluster. To understand the consistencies between our dataset and other urinary datasets, a previously published kidney single‐cell dataset was downloaded and analysed.^32^

### 2.9 Intercellular crosstalk analysis

To explore potential intercellular crosstalk between SOX9+ RECs and other cell types from healthy control (HC) and AKI samples, we implied the ligand‐receptor distribution and expression of SOX9+ RECs and other cell types with a standard pipeline implemented in R using CellChat R package,^33^ as previously reported. We chose the receptors and ligands expressed in more than 10% of the cells in the specific cluster for subsequent analysis. The interaction pairs whose ligands belonged to the epidermal growth factor (EGF), vascular endothelial growth factor (VEGF) and Complement families were selected for the evaluation of intercellular crosstalk between the SOX9+ RECs and other cell types.

### 2.10 Proteomic analysis

Protein extraction and pre‐treatment of enzymolysis and desalination at Novegene, China. Then polypeptides were labelled by Tandem Mass Tags. Subsequently, combining the polypeptides, fractionate, clean up and perform QE HF‐X LC–MS/MS analysis. Raw sequencing data were processed and quantitated using Proteome Discoverer Software 2.2. Six serum samples divided into three groups were analysed. The differential expression of each two groups was performed using the DESeq2 R package. Due to poor sample repeatability, some protein data with high Foldchange values were deleted, and then the remaining data were averaged. The protein dataset had been normalized by the housekeeping gene GAPDH to reduce the extraneous variation among samples. PCA analysis was performed using the gmodels R package (version 2.18.1) and visualized by the ggthemes R package (version 4.2.4). Heatmap showed the average expression of three groups. Ward's minimum variance method was used and the result was visualized by using the ggplots R package (version 3.1.1). Protein–protein interaction (PPI) network was constructed to map the differentially expressed genes (DEGs) to the protein data by using Cytoscape (3.9.0).^34^

### 2.11 Statistical analysis

Results were expressed as means ± SD. Matched results were assessed by two‐way ANOVA. Comparisons among multiple groups were analysed using Tukey's multiple comparison test. GraphPad Prism (version 7.0a) or R programming was used for data management, quantitative analysis and graph generation. Differences with *p* ≤ 0.05 were considered statistically significant.

## 3 RESULTS

### 3.1 Activation of SOX9+ RECs in injured kidneys of mouse models

**FIGURE 1:**
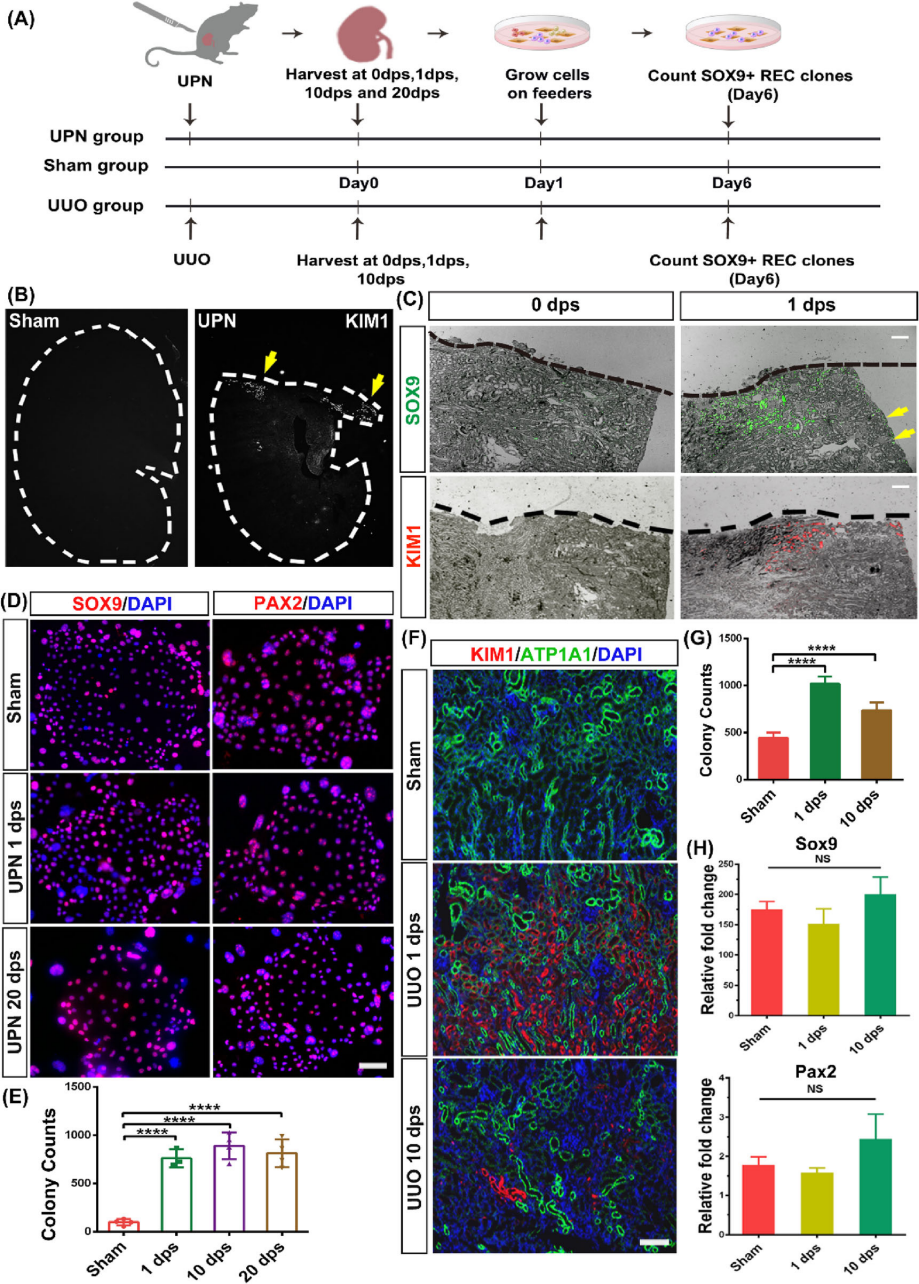
SOX9+ REC activation in injured mouse kidneys. (A) Establishment of the mouse UPN and UUO injury model and subsequent cloning of SOX9+ RECs from sham and injured kidneys. (B) The mouse's left kidney was partially nephrectomized on the upper 1/3 part. Yellow arrowheads indicated KIM1+ cells by immunostaining. (C) Immunofluorescent staining of SOX9 and KIM1 in the UPN injured kidney on different dps. Black dashed lines indicate the blade cut edge. Yellow arrowheads indicate SOX9‐positive RECs, which were activated away from the incision. Scale bar, 500 μm. (D) REC colonies derived from sham and UPN injured kidneys immunostained with SOX9 and PAX2 antibodies. Scale bar, 50 μm. (E) The colony counts derived from sham and partial nephrectomy injured kidneys (*n* = 4 mice). (F) Immunofluorescence labelling of injured kidney in a UUO model with antibodies against KIM1 and ATP1A1 to identify damaged tissue and healthy proximal tubules, respectively. (G) The colony counts derived from sham and UUO injured kidneys (*n* = 3 mice). (H) RT‐qPCR analysis of transcripts of *Sox9* and *Pax2* in RECs derived from sham and UUO injured kidneys. (*n* = 3 independent RT‐qPCR experiments). Statistics were inclusive of all biological replicates. Data were presented as means ± SD.****p < 0.0001.

Previous *in vivo* lineage tracing studies have indicated a strong correlation between SOX9+ cell activation and early transcriptional response to murine renal injury.^9^, ^17^ Here we introduced a new UPN mouse model to confirm the process as depicted in Figure 1A. For normal mice kidneys, we detected 0.051(±0.016) % and 0.021(±0.018) % cells were positive for SOX9 staining in the cortex and medulla region, respectively. One day post‐UPN surgery (1 dps), we observed strong expression of kidney injury marker (KIM1) along the cutting edge of the cortex (Figure 1B) and simultaneous emergence of a large number of SOX9+ REC surrounding KIM1 positive tubular area (Figures 1C and S1A). Unexpectedly, a fraction of SOX9‐positive, as shown by yellow arrows, were also activated away from the SOX9+ cell niche and scattered along the outer edge of the cortex (Figure 1C, arrows).

Next, we tried to dissociate kidney tissues harvested at multiple dps and plated dissociated cells using a feeder‐based regenerative cloning (R‐Clone) culture system.^35^, ^36^, ^37^, ^38^ The activated mouse cells obtained from UPN kidneys, as those from normal kidneys, represented similar clonogenicity (Figure S1B) and stained positive for SOX9 and PAX2, which are acknowledged markers of nephron progenitors^39^ (Figure 1D). Consistent with the *in vivo* immunostaining data, the kidney tissue subjected to UPN yielded approximately five‐fold more clones, which surged as early as 1 dps (Figure 1E).

We also examined whether similarly activated RECs can be cloned in other kidney injury models with a different damage mechanism. The UUO injured model could also induce substantial tissue damage in the cortex and medulla regions,^40^ which was characterized by tubule marker (ATP1A1+) loss and KIM1 expression (Figure 1F). As with the UUO injury, the time‐course clonogenic assay demonstrated more SOX9+ RECs 1 day after UUO injury (Figure 1G). The seemingly identical staining and RT‐qPCR expression levels of *Sox9* and *Pax2* suggested that the activated RECs shared similar biological characteristics with resident SOX9+ RECs under normal conditions (Figures 1H and S1C). Therefore, all these data suggested endogenous SOX9+ RECs could be stimulated to expand post different types of renal injury.

### 3.2 Activation of SOX9+ RECs in injured human kidney

**FIGURE 2:**
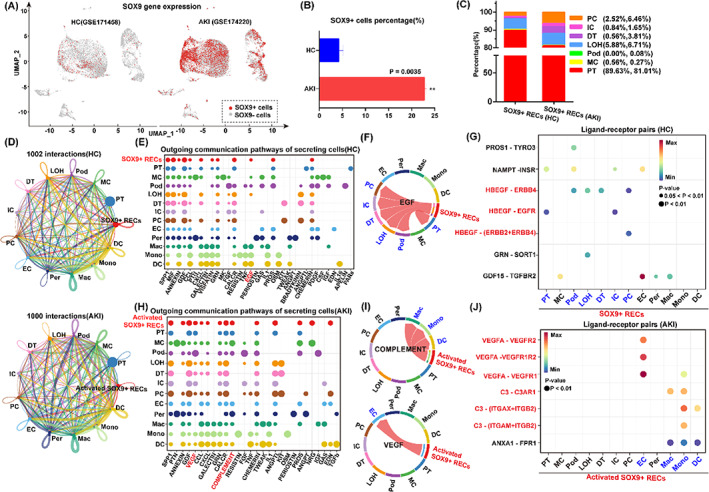
Intercellular crosstalk analysis of SOX9+ RECs in human kidneys. (A,B) Visualization (A) and quantification (B) of SOX9+ cells in HC (GSE171458) and AKI (GSE174220). Data were presented as means ± SD.**p < 0.01. (C) Cumulative analysis represented the distribution of SOX9+ RECs in different subjects. Podocytes (Pod), proximal tubule cells (PT), Loop of Henle (LOH), distal cells (DT), mesangial cells (MC), intercalated cells (IC) and principle cells (PC). (D) Global intracellular communication network of 13 cell groups in HC and AKI samples. The thicker the line represents, the more the number of interactions. Different colours in the circle plot show different cell groups and circle sizes are proportional to the number of cells in each cell group. Endothelial cells (EC), pericytes (Per), macrophages (Mac), monocytes (Mono) and dendritic cells (DC). (E) The dot plot showed the outgoing signalling pathways of secreting cells in HC. The dot size is proportional to the contribution score. A higher contribution score implies the signalling pathway is more enriched in the corresponding cell group. (F) Visualization of the inferred communication network of EGF signalling pathway in HC group. In the Chord diagram, the inner thinner bar colours represent the targets (blue) that receive the signal from the SOX9+ RECs (red). The inner bar size is proportional to the signal strength received by the targeted groups. (G) Analysis of the significant ligand‐receptor pairs in HC group, which contributed to the EGF signalling from SOX9+ RECs (red) to other cell populations. The dot colour reflects communication probabilities and the dot size displays the computed *p*‐values. Empty space means the communication probability is zero. *p*‐values are computed from one‐sided permutation test. (H) The dot plot exhibited the outgoing signalling pathways of secreting cells in AKI. Larger size of the dot plot reveals the signalling pathway is more enriched in the corresponding cell group. (I) Visualization of the inferred communication network of signalling pathways in AKI group from the activated SOX9 + RECs (red) to the targets (blue), including COMPLEMENT and VEGF. (J) Interactions of the significant ligand‐receptor pairs contributed to the inferred signalling from activated SOX9+ RECs (red) to other cell populations (blue) in AKI.

Previous studies by our group and others^9^, ^10^, ^17^, ^41^ indicated activation of SOX9+ cells in injured rodent kidneys for tissue repair purposes, however whether a similar mechanism is applied to humans remained unknown. To investigate the existence of tissue resident SOX9+ populations, human kidney sc‐RNA‐seq datasets (GSE171458; GSE174220) obtained from two healthy controls (HC) and two patients with AKI were analysed.^42^ After data pre‐processing and stringent quality control, 12 clusters from four different kidney subjects were visualized by UMAP (Figure S2A,B) and the selected cell lineage‐specific marker gene was displayed by dot plot (Figure S2C). As expected, we found SOX9+ cells in renal tissues (Figure 2A). There were about 4.217 ± 0.987% cells expressing SOX9 in the HC group, while the percentage of SOX9+ RECs significantly increased up to 22.71 ± 0.478% in the AKI group (Figure 2B), suggesting the activation of SOX9+ cells in injured human kidneys. Of note, all SOX9+ cells were restricted to epithelial‐related clusters. Hereafter, we referred to these SOX9+ cells as RECs. In either healthy or AKI kidneys, both SOX9+ cells were concentrated in multiple segments of tubular cells (Figure 2C).

Gene Ontology (GO) function analysis showed that the differentially expressed genes of SOX9+ RECs were associated with not only renal development, but also the secretory regulation processes, manifested by genes including *CD24, HES1, PAX2, HBEGF and VEGF* (Figure S2D). Therefore, we focused our studies on the secretory regulation of SOX9+ RECs on kidney repair. To further identify potential cell–cell interactions between SOX9+ RECs and other cell groups, we performed ligand‐receptor analysis with the CellChat package. CellChat detected over 1000 significant interactions among the 13 cell groups in HC and AKI samples (Figure 2D). While the number of interactions was nearly unchanged, the contribution of outgoing communication pathways varied in the healthy or injured states.

In HC group, CellChat detected 61 significant ligand‐receptor pairs in the SOX9+ RECs group, which were further categorized into 12 signalling pathways (Figure 2E). Network centrality analysis of the signalling network identified the EGF pathway as the dominant interaction between the SOX9+ RECs and other adjacent epithelium groups in health status, including podocytes (Pod), proximal tubule cells (PT), Loop of Henle (LOH), distal cells (DT), intercalated cells (IC) and principle cells (PC) (Figure 2F). Further ligand‐receptor pairs analysis revealed that the growth factor signal axis such as HBEGF‐ERBB1, HBEGF‐EGFR and HEEGF‐(ERBB2 + ERBB4) might participate in the intercellular crosstalk, which was critical for the regulation of normal epithelial homeostasis (Figure 2G).

Under the damaged condition, the categorized pathways of activated SOX9+ RECs included VEGF, COMPLEMENT, SPP1 and CALCR (Figure 2H). We detected COMPLEMENT and VEGF signalling pairs as the dominant pathways in AKI kidneys. The Network centrality analysis revealed that COMPLEMENT was more enriched between activated SOX9+ RECs and immune cells, especially macrophages (Mac) (Figure 2I). The kidney resident macrophages were predominantly functional on activation of the complement system,^43^ demonstrating a protective role under the injured state.^44^ Meanwhile, VEGF is a crucial factor contributing to angiogenesis.^30^ Our data showed the VEGF signalling pathway had closely interacted between activated SOX9+ RECs and endothelial cells (EC) (Figure 2I). To validate these inferred interactions, we performed further ligand‐receptor analysis. The bubble plot revealed that activated SOX9+ RECs in AKI expressed C3 with receptors expression by macrophage (Mac), monocytes (Mono) and dendritic cells (DC). Besides, we also identified the close interactions between VEGFA and their receptors VEGFR1, VEGFR2 and VEGFR1R2 expressed by ECs (Figure 2J). Altogether, the data above showed that the SOX9+ REC could be activated and expanded in injured human kidneys, and secreted multiple factors to facilitate kidney repair.

### 3.3 Identification of SOX9+ cells in urine from humans of different ages

**FIGURE 3:**
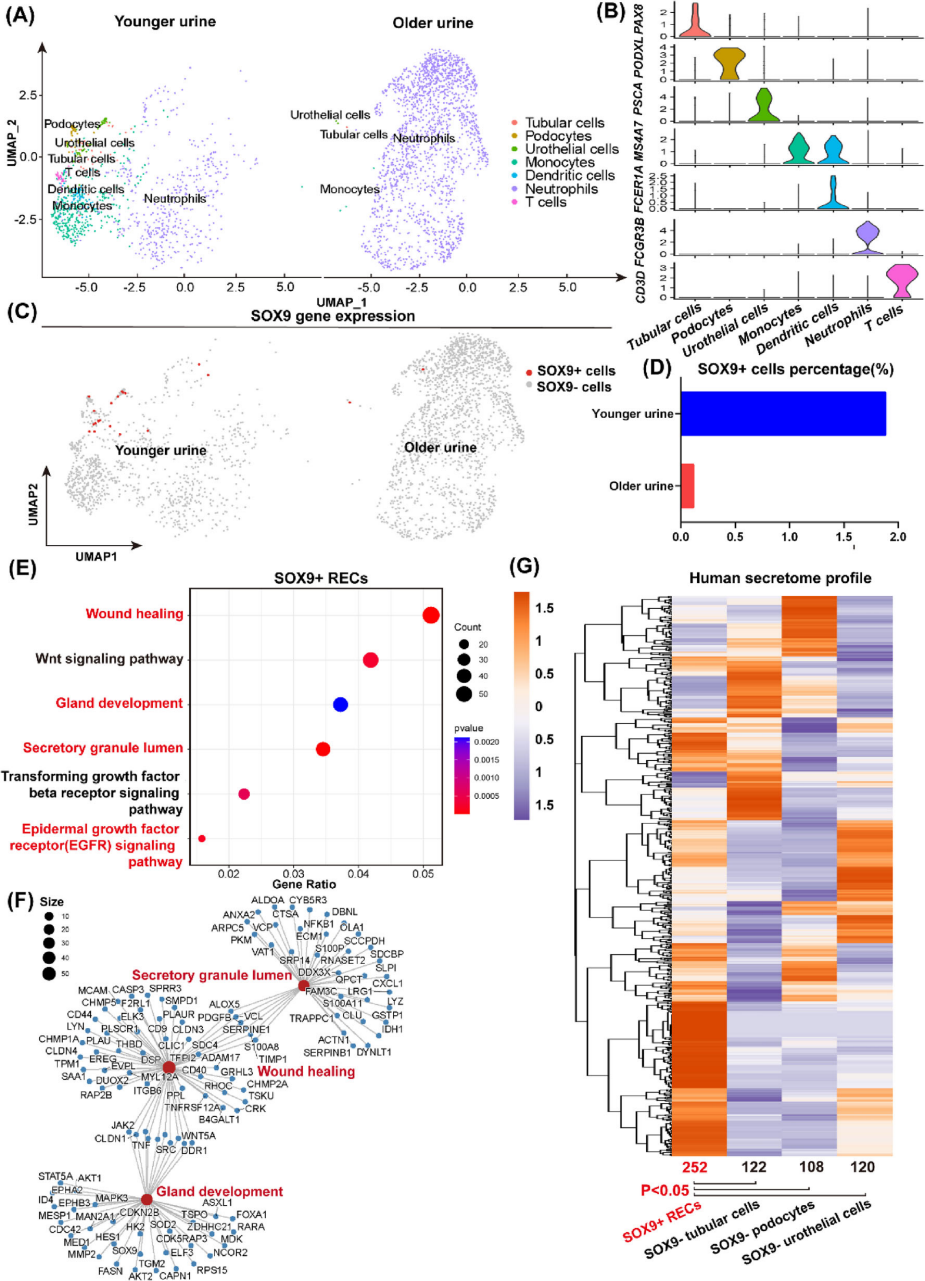
Characterizations of urinary SOX9+ cells. (A) UMAP visualization of cell fractions of healthy urine from people of different ages. (B) Violin plots showed representative marker genes across seven clusters. The y‐axis shows the log‐scale normalized read count. (C,D) Visualization (C) and quantification (D) of *SOX9* gene expression indicated a higher percentage of SOX9+ cells the younger group compared to the older group. (E) GO and KEGG enrichment analysis of genes highly expressed in SOX9+ RECs. (F) Network visualization of genes with high expression levels of inferred GO terms. (G) Heatmap representation of gene expression for human secretome dataset among four groups. The number and average expression level of the enriched gene set were compared between SOX9+ RECs and other SOX9− groups. Results were accessed by unpaired *t*‐test. *p < 0.05.

In previous studies, we reported the single‐cell atlas of human urine.^29^ Here we focused on the kidney repair‐related SOX9+ RECs and analysed them in the urine samples collected from younger (*n* = 12, median age = 24) and older (*n* = 40, median age = 49) healthy people. In total, we sequenced 1104 cells from the younger age group and 2386 cells from the older age group. After stringent quality controls, we eventually analysed 1010 and 1774 cells, respectively. Our clustering indicated seven clusters under unsupervised graph‐based clustering (Seurat method) of the dataset and visualization by UMAP (Figure 3A). We annotated the identity of the clusters from two groups based on the established cell type‐specific markers. Violin plots for representative differentially expressed genes from each of the populations were shown, including tubular cells (*PAX8*+), podocytes (*PODXL*+), urothelium cells (*PSCA*+) and immune cells (monocytes, dendritic cells, Neutrophils and T cells) (Figure 3B). Compared to the younger urine, we found that neutrophils were the most abundant cell population in the older urine, accounting for 95% of total cells, which could be an indicator of aging related kidney inflammation.^45^

SOX9+ cells were found in both younger and older urine samples (Figure 3C). The percentage of SOX9+ cells was 10‐fold higher in the younger group (1.9%) in comparison with the older group (0.11%), suggesting an age‐related decline of renal homeostasis (Figure 3D). By analysing the distribution of cell types in SOX9+ cells, we found that 62.5% of them were urothelial cells, while others were from tubular cells (18.75%) or podocytes (18.75%). In addition to our sc‐RNA‐seq data, we also analysed another published human urine single‐cell RNA sequencing dataset (GSE157640) and obtained similar results (Figure S3A,B).

GO and Kyoto Encyclopedia of Genes and Genomes (KEGG) enrichment analysis demonstrated that the differentially expressed genes identified urinary SOX9+ RECs enriched in specific processes, including wound healing, gland development, secretory granule lumen and epidermal growth factor receptor (EGFR) signalling pathway (Figure 3E). Network analysis showed that the secretory granule lumen related genes are closely linked to the wound healing process (Figure 3F). To further study the secretion related gene expression in SOX9+ REC, we compared our sc‐RNA‐seq data with a curated human secretome dataset with 2641 known genes.^46^ Hierarchical clustering enriched 602 genes totally in our dataset, among which 252 secreted genes were highly expressed in SOX9+ RECs, such as *S100A9*, *MUC1* and *NOTCH2*. To compare the secretory capability of different groups, we calculated the average expression level of the gene set in each group. We found that SOX9 + RECs demonstrated significantly higher expression for secretome profiles compared to SOX9‐ cell groups (*p* < 0.05) in our dataset (Figure 3G) and others (Figure S3C). Altogether these data indicated that a rare population of SOX9+ RECs existed in human urine, which showed extraordinary secretory capability.

### 3.4 Long‐term, feeder‐free culture of urine‐derived SOX9+ RECs

**FIGURE 4:**
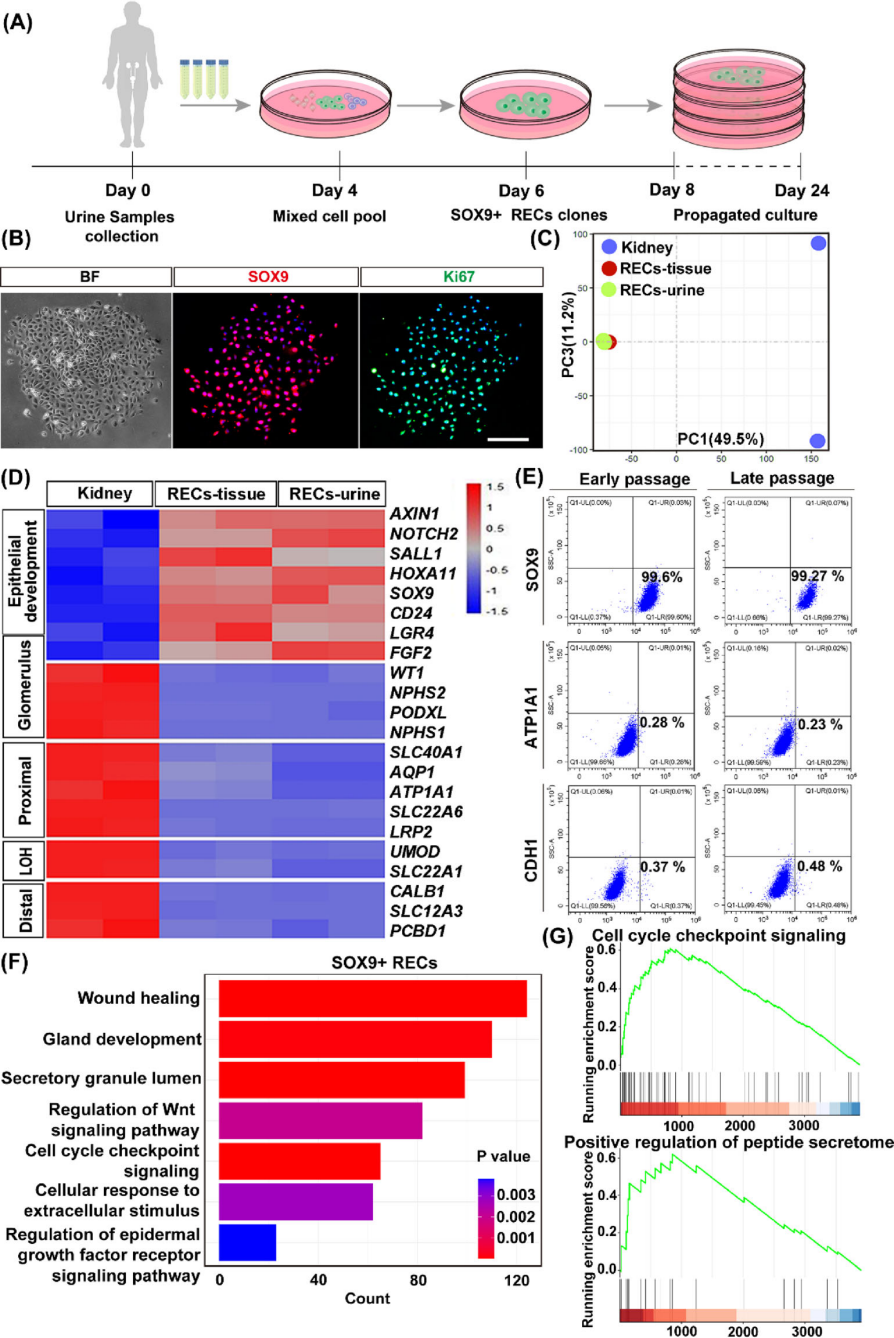
Long‐term cloning of human SOX9+ RECs. (A) Schematic showed the selective culture of RECs from human urine specimens and propagated culture for a long time. (B) Human RECs stained with SOX9 antibody and proliferative marker Ki67. Scale bar, 100 μm. (C,D) PCA and Heatmap of differentially expressed gene set exhibited a distinct transcriptome of kidney tissues and cultured SOX9+ RECs from renal tissue (RECs‐tissue) and urine (RECs‐urine). Duplicates were taken from independent biological samples. (E) Flow cytometry analysis on the long‐term cultured RECs as assessed by indicated markers SOX9, ATP1A1 and CDH1 (*n* = 3 independent experiments in each group). (F) GO enrichment analysis of differentially expressed genes in cultured SOX9+ RECs. (G) GSEA plots demonstrated the running enrichment score of gene sets in the bulk RNA data of cultured SOX9+ RECs.

Next, we try to culture the human SOX9+ RECs for further studies. Our previous work described the successful cloning of the SOX9+ RECs from patient renal tissue biopsy using a murine feeder‐cell based system (also demonstrated in Figure S4A).^29^ Here we further improved the method and successfully cloned the urine‐derived SOX9+ RECs in a feeder‐free culture system (Figure 4A), which allowed the xeno‐free mass production of cells in the future for therapeutic purposes. Expanded cells from urine (RECs‐urine) shared consistent morphology and proliferative (SOX9+, Ki67+) characteristics with cells from kidney tissue biopsy (RECs‐tissue) by immunostaining (Figures 4B and S4A). Transcriptome analysis further confirmed such RECs‐urine showed great similarity to RECs‐tissue, with high expression of a small number of genes related to epithelial development, such as *SOX9*, *CD24*, *NOTCH2* and *SALL1*,^47^, ^48^ whereas hardly expressed mature glomeruli and tubules markers (*LRP2*, *ATP1A1*, *AQP1*, *UMOD* and *SLC12A3*) (Figure 4C,D).

To further characterize the cells after continuous culture, SOX9+ RECs were propagated for >8 passages. Quantitative flow cytometry analysis showed a high percentage of SOX9 expression (99.6% at the early passage P2; 99.27% at the late passage P8) during the culture process and a low percentage of mature epithelial markers (ATP1A1+ or CDH1+; Figure 4E). The mature epithelial gene expression profile of RECs was also confirmed by immunostaining, highlighting no dramatic differences in different passages of RECs (Figure S4B). Finally, GO enrichment analysis of cultured SOX9+ RECs presented extensive genes linked to cell proliferation, wound healing and secretory regulation related terms (Figure 4F), and especially related to cell cycle checkpoint signalling and positive regulation of peptide secretome (Figure 4G). Altogether the data showed the possibility of long‐term, feeder‐free culture of SOX9+ RECs while maintaining their cell properties, which provides an opportunity for future functional research and cell therapy translational application.

### 3.5 SOX9+ REC‐derived secretory factors facilitated the kidney repair process

**FIGURE 5:**
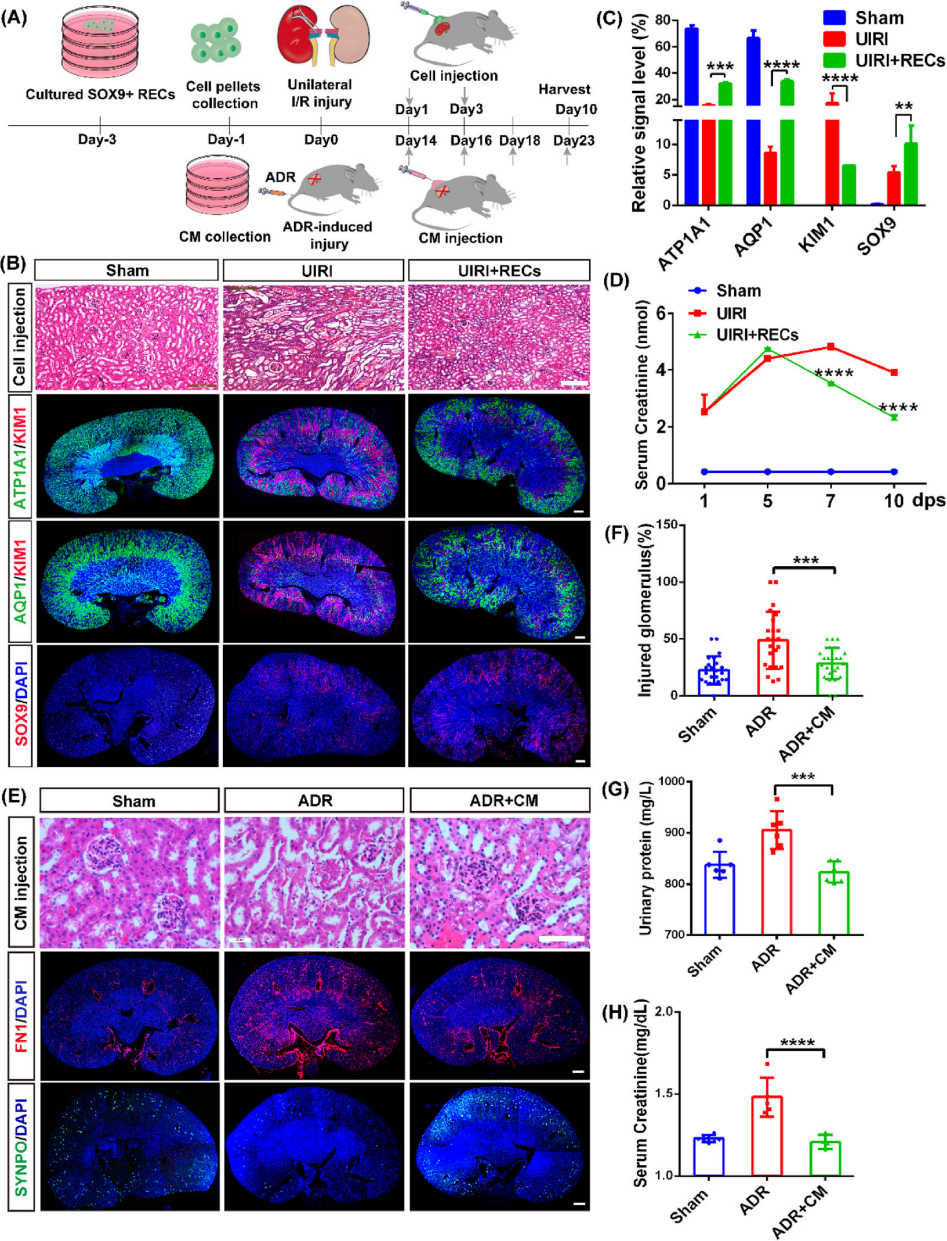
Therapeutic effects of SOX9+ REC secretome in kidney injury models. (A) Schematic illustrated the actual REC‐dependent/ independent treatments after mice injury by subcutaneous injection. (B) Representative images of H&E and immunofluorescent staining (IF) revealed the regeneration of renal tubules after REC treatment. Sham, no surgery; UIRI, 3T3 cells pellets subcutaneous injection containing 2–4^10^6^ after UIRI; UIRI+ RECs, REC pellets subcutaneous engraftment with equal number of cells after UIRI. Scale bar, 100 μm (HE); Scale bar, 200 μm (IF). (C) Quantification of the relative signal level after REC therapy. (D) Serum creatinine level measurement at the indicated checkpoint after REC treatment. (E) Representative H&E and IF indicated the recovery of glomerulus epithelium post CM injection with a dose of 2 mg secretome proteins. Sham, no surgery; ADR, DMEM subcutaneous injection with 200 μl/per after 10.5 mg/kg ADR administration; ADR + CM, equivalent‐volume CM injection after ADR injury. Scale bar, 20 μm (HE); Scale bar, 200 μm (IF). (F) The proportion of injured glomerulus was accessed semi‐quantitatively by point counting. Each evaluation was mean ± SEM (*n* = 25 views per group). (G,H) Urinary protein and serum creatinine levels after CM therapy. Data shown in (C), (D), (F), (G) and (H) were represented as mean ± SD (**p < 0.01, ***p < 0.001, ****p < 0.0001, *n* = 3 independent biological samples, each group made in duplicate).

Obtaining of SOX9+ RECs in culture makes it possible to study whether the cell could contribute to kidney repair through secretory mechanisms. To address this question, a murine model of renal UIRI model was established. As the previous experiments reported, mice with surgery demonstrated severe kidney damage,^49^, ^50^ and the activation of SOX9+ RECs started within 24 h post injury.^9^, ^17^ Then we subcutaneously engrafted RECs since 1 day after renal damage and collected the data until day 10 (Figure 5A). The data showed that the UIRI resulted in remarkable tubular epithelial necrosis, cellular debris accumulation and renal tubule dilation, whereas these damages were ameliorated by subcutaneous (S.C.) REC engraftment (Figures 5B and S5A). To fully assess the efficacy of REC treatment, the whole kidney sections were stained with anti‐KIM1, anti‐ATP1A1 and anti‐AQP1 antibodies for the detection of injured and healthy tubule areas. REC S.C. treatment reduced the KIM1+ damaged area, accompanied by reconstitution of ATP1A1+ and AQP1+ renal tubular epithelial compared with the UIRI group. Then, we noted that the acute injury stimulated partial SOX9 activations while S.C. REC engraftment treatment significantly increased the number of renal resident SOX9+ cells (Figure 5B,C). Moreover, evaluation of renal function in UIRI mice demonstrated REC treatment reduced serum creatinine levels by day 7 and maintained until day 10 (Figure 5D).

We also evaluated the therapeutic efficacy of SOX9+ RECs through the intraperitoneal (I.P.) injection route. Interestingly, we found that the I.P. injected GFP‐positive cells distributed at the surface of the unilateral injured kidney on the right side selectively, whereas no cells resided in the contra‐laterally healthy kidney (Figure S6C). Similar to the S.C. injected cells, the I.P. injected cells showed consistent therapeutic effects in terms of tubule histological improvement and functional restoration (Figure S6A–F).

To further confirm the function mechanism without actual cells, we collected supernatant of cultured RECs as a CM. The CM was allowed to condition for 1 day before it was harvested and centrifuged at 1000*g* for 15 min to remove any dead cells and cell debris. By S.C. injection of CM, we observed consistent therapeutic effects with less injured KIM1+ tubules, increased expressions of healthy proximal tubule markers (ATP1A1+/AQP1+), activated more endogenous SOX9+ cells and decreased the serum creatinine levels (Figure S7A–D). We also found that CM treatment could significantly enhance the proliferative capability of cultured SOX9+ RECs *in vitro* (Figure S7E), suggesting the self‐activation of cells by an autocrine mechanism.

Furthermore, we examined whether the SOX9+ REC‐derived secretory factors would affect the repair of the glomerulus in an ADR‐induced glomerular injury model. The results indicated that the ADR intravenous (I.V.) injection resulted in remarkable glomeruli tuft and glomerular adhesion of the renal capsular membrane, whereas these damages were ameliorated by S.C. injection of REC‐derived CM like the previous UIRI study (Figure 5E). Histologic examination displayed the number of injured glomeruli and necrosis scores of tubules with a significant reduction in the CM treatment group compared with the ADR group (Figures 5F and S5B). To fully evaluate the efficacy of CM treatment, the whole kidney sections were stained with anti‐fibrotic marker Fibronectin (FN1) and anti‐SYNPO antibodies. S.C. CM treatment reduced the FN1+ fibrotic area, accompanied by recovery of SYNPO+ podocytes in the glomeruli compared with the ADR group (Figure 5E and S5C). Consistent with the histological restoration, both the urinary protein and serum creatinine level showed significant decrease in mice receiving CM administration compared with the ADR group (Figure 5G,H). Taken together, these findings demonstrated that the secretory factors of SOX9+ RECs could simulate endogenous SOX9+ cell self‐activation, attributing to the regeneration of renal tubules and glomeruli.

### 3.6 Quantitative proteome analysis of SOX9+ REC engrafted mice

**FIGURE 6:**
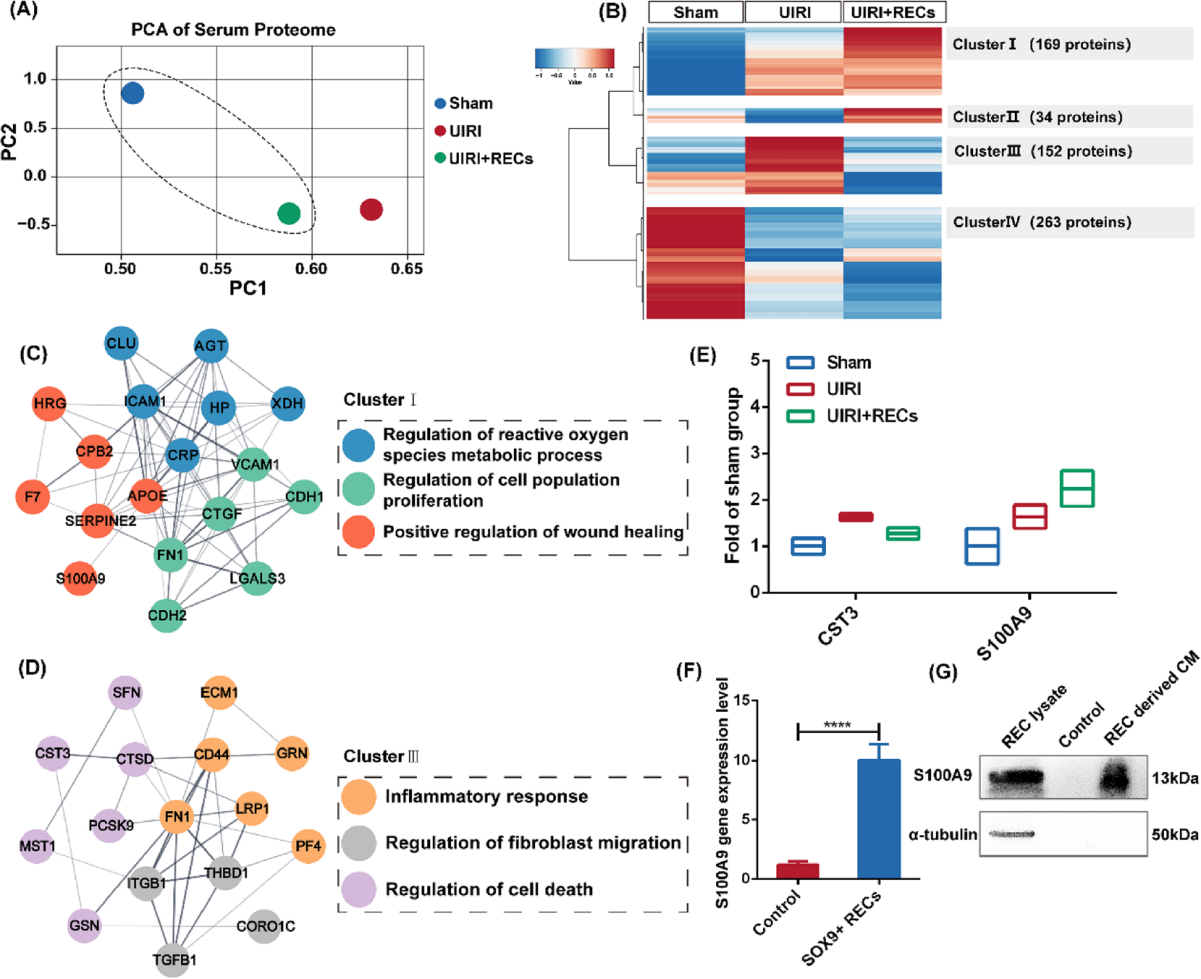
Quantitative proteomic analysis of SOX9+ REC transplanted mice. (A) PCA of Tandem mass tags (TMT) proteome data in three groups (Average *n* = 3 independent biological samples). Different colours represented distinct groups. UIRI, mice injured by unilateral ischemia–reperfusion injury; UIRI + RECs, mice subcutaneously treated by 2–4^10^6^ RECs after UIRI. (B) Heatmaps presented the four different tendencies after REC subcutaneous injection therapy. Six hundred eighteen proteins were significantly changed (*p*‐value < 0.05) among the three groups. Data were scaled across rows before mapping to colours. (C,D) PPI network analysis of the expression of proteins associated with specific GO terms and their interaction relationship in Cluster I (C) and Cluster III (D). (E) Quantitative analysis of selected factors after REC administration. (F) Relative mRNA expression was quantified by RT‐qPCR, indicating higher expression of *S100A9* in cultured SOX9+ REC line than *in vivo* (control). (*n* = 3 independent RT‐qPCR experiments). Statistics were inclusive of all biological replicates. (G) Detection of secreted S100A9 expression in the SOX9+ REC‐derived CM. Control, DMEM media.

To identify the secreted proteins involved in renal injury and repair, we applied a proteomic strategy to study the blood serum of SOX9+ REC engrafted mice. We used a multiplexed quantitative proteomics approach following tandem tag‐based mass spectrometry, a technology that enables protein identification and quantitation from multiple samples simultaneously.^51^, ^52^, ^53^ Multiplexed quantitative proteomic analyses were performed using blood serum samples from the sham operation, UIRI and UIRI + RECs groups. In this unbiased global proteomics screening platform, a total of 682 proteins were quantified across three different serum samples. As visualized in the PCA clustering results (Figure 6A), the PC1 axis, as the first principal direction, showed a smaller difference between UIRI + RECs versus Sham groups than UIRI versus Sham groups, which demonstrated that the secretome treatment could moderate the changes from renal injury.

To further systematically assess REC secretome treatment induced changes and identify proteins of interest, we subjected the proteomic datasets to hierarchical clustering based on Ward's minimum variance method,^54^ which was visualized in a heatmap. Four distinct abundance profiles were recognized: Cluster I comprised 169 secreted proteins that gradually increased in abundance, reaching a maximum in the REC treatment group; Cluster II involved 34 proteins whose level decreased before growing; Cluster III contained 152 proteins presented inverse tendency with Cluster II; Finally, Cluster IV involved 263 proteins that continuously decreased in abundance over different groups (Figure 6B). The GO of each cluster was analysed in Figure S8, showing that the Cluster I proteins were closely related to wound healing while Cluster III proteins were related to neutrophil immune response. More specifically, our proteome data allowed us to localize the expression of genes associated with specific pathways and illustrate their interaction relationship by PPI network analysis. The results indicated that the REC treatment group highly expressed many biological processes component genes of Cluster I, which formed an interaction network about ‘Regulation of reactive oxygen species metabolic process’, ‘Positive regulation of cell proliferation’ and ‘Positive regulation of wound healing’ (Figure 6C). In contrast, the UIRI group highly expressed genes (Cluster III) which formed an interaction network about ‘Inflammatory response’, ‘Regulation of fibroblast migration’ and ‘Regulation of cell death’, which were all involved in tissues injury status (Figure 6D). Therefore, we speculated that there were deleterious factors in Cluster III while Cluster I probably contained beneficial proteins for the kidney.

Indeed, among these proteins in clusters, we noticed a few interesting candidates for further analysis. Cystatin‐C (CST3) protein in Cluster III is known as a critical biomarker for monitoring renal toxicity or harm in clinical trials,^55^, ^56^, ^57^ which was increased in expression in our datasets after UIRI and inversely correlated with recovery post REC engraftment treatment (Figure 6E). In contrast, S100 calcium‐binding protein 9 (S100A9) increased in the UIRI group and continuously increased after REC treatment (>2.2‐fold) (Figure 6E). As mentioned in the above text, we have identified *S100A9* overexpression in the sc‐RNA‐Seq data of urine‐derived SOX9+ RECs, which was confirmed here in cultured SOX9+ RECs by RT‐qPCR (Figure 6F). S100A9 protein was also detected in CM from SOX9+ RECs (Figure 6G). Therefore, it is very likely that SOX9+ RECs could promote kidney repair at least partially by secreting S100A9 protein.

### 3.7 Recombinant S100A9 protein promotes renal tissue repair after AKI

**FIGURE 7:**
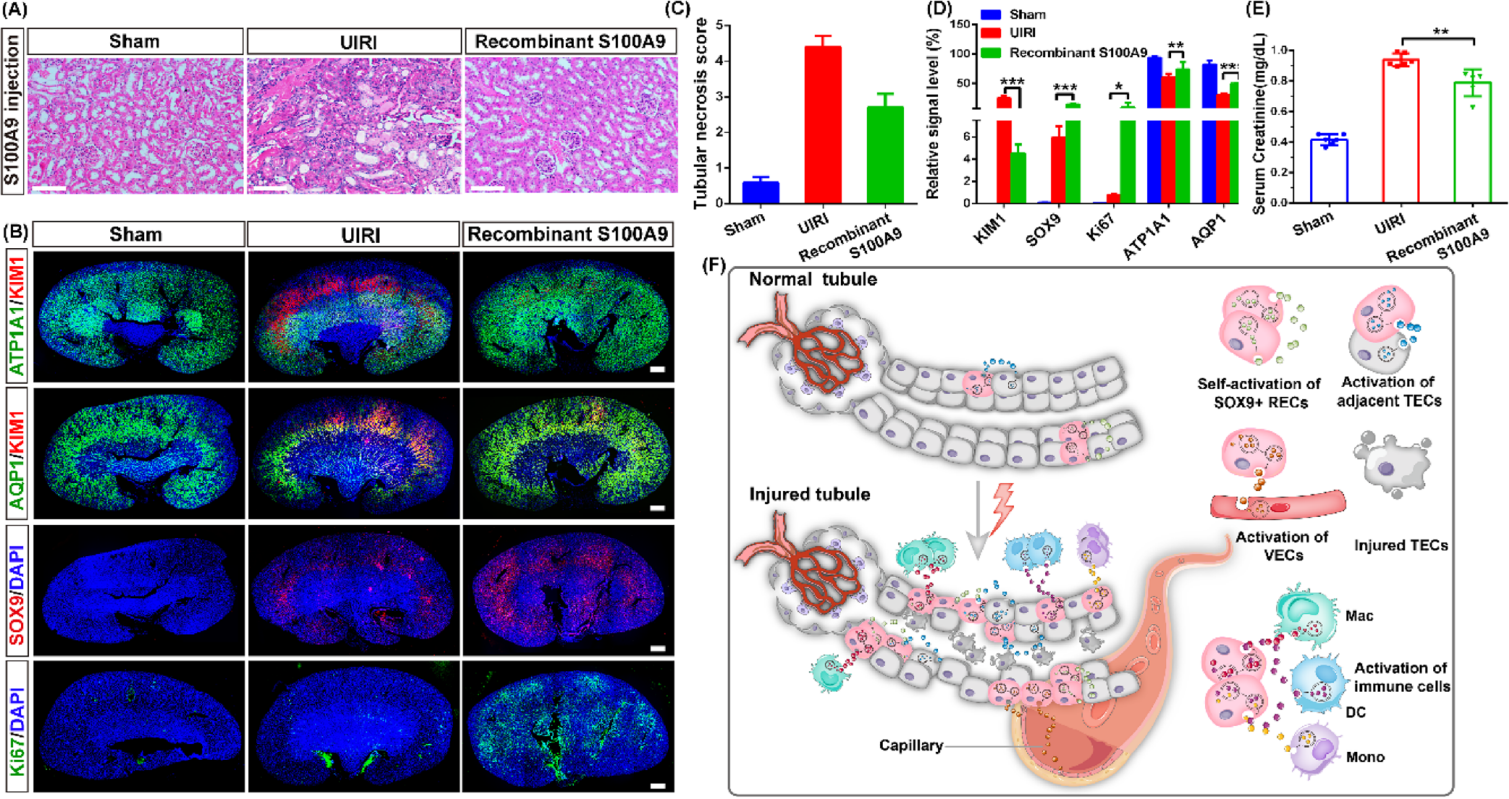
Recombinant S100A9 protein ameliorates renal restoration. (A) Representative image of H&E‐stained indicated less tubular injury post recombinant S100A9 intervention for 7 days. Sham, with no surgery; UIRI, an equal volume of saline subcutaneous injection since 1 day after UIRI; recombinant S100A9, twice, 10ug/kg recombinant S100A9 protein subcutaneous administration. Scale bar, 50 μm. (B) Representative images of the immunostained whole kidneys after recombinant S100A9 protein treatment. Scale bar, 200 μm. (C) Quantitative scores of tubular necrosis. (D) Quantification of KIM1, ATP1A1, AQP1, SOX9 and Ki67 gene expressions after UIRI or recombinant S100A9 treatment. (E) Serum creatinine level decreased after recombinant S100A9 treatment. Data shown in (C), (D) and (E) were represented as mean ± SD (*p < 0.5, **p < 0.01, ***p < 0.001, *n* = 3 independent biological samples per group, each group made in duplicate). (F) Assumptive model of SOX9+ REC‐driven tubular restoration under injured triggers by secretome. S100A9 protein activates resident SOX9+ RECs proliferation. EGF, VEGF and complement signallings from SOX9+ RECs promote renal repair by acting on the adjacent TECs, VECs and immune cells. The components of SOX9+ REC secretome is represented by colourful graphics. TECs, tubular epithelial cells; VECs, vascular endothelial cells.

S100A9 is a member of the S100 calcium‐binding protein family. S100A9 deficiency displayed a phenotype with enhanced renal damage, revealing that S100A9 could contribute to kidney repair.^58^ To further predict the role of S100A9 in AKI, we monitored the reduction in renal function following renal UIRI and the effects of the human recombinant S100A9 protein administration with twice, high‐dose, S.C. and I.V. injection. The results showed that the kidneys exhibited severe morphological injury post UIRI, including cellular debris accumulation, and renal tubular dilation and cell apoptosis. While recombinant S100A9 injection significantly attenuated tubular injury, which was demonstrated by H&E and immunofluorescent staining with antibodies (KIM1 for injured tubule, ATP1A1 and AQP1 for healthy tubule) (Figures 7A–D and S9A–C).

Moreover, we observed that a few SOX9+ RECs appeared after UIRI and the number of SOX9+ RECs was significantly increased after the recombinant S100A9 protein treatment. Interestingly, after S100A9 protein administration, the kidney showed a high proliferation ratio (10% Ki67+) *in vivo*, which could be related to the regeneration of the proximal tubular epithelium (ATP1A1+ and AQP1+) (Figures 7B,C and S9B). We also validated the pro‐proliferative effect of S100A9 on SOX9+ RECs *in vitro* (Figure S9E). Mice injected with exogenous S100A9 protein showed a significant reduction in serum creatinine level compared with the UIRI group, suggesting that exogenous S100A9 can restore renal injury in both morphology and function (Figures 7E and S9D). Taken together, the data showed that S100A9 is one of the bioactive factors, which could trigger resident SOX9+ REC proliferation. And other components in the secretome could also participate in regeneration, partially by activation of EGF, VEGF and Complement signal pathways. (summarized in Figure 7F).

## 4 DISCUSSION

In the current study, we noted that the endogenous SOX9+ RECs could expand in the acutely damaged kidney of both mice and human. At the single‐cell resolution, the SOX9+ RECs displayed the dominantly secreted signature, which was closely linked to kidney regeneration pathways. Based on a feeder‐cell‐independent regenerative cloning (R‐Clone) system, we successfully obtained the cultured SOX9+ RECs from human urine, maintaining stable cell characteristics and secretory function. Transplantation of cultured human SOX9+ RECs or administration with its CM ameliorated renal tubular and glomerular epithelium damage. We also confirmed S100A9 as one of the critical factors in the SOX9+ REC secretome by quantitative proteome analysis and functional studies.

The isolation and expansion system of epithelial cells from adult tissues are fundamental techniques supporting cell‐based regeneration studies. In 1975, Green et al. established the first successful example of adult human epidermal stem cell culture.^35^ Our previous studies have obtained SOX9+/Ki67+ populations from human urine with massive animal‐derived components, relying on a mouse fibroblast feeder layer.^29^ Here we improved the method and cultured SOX9+ RECs in a novel feeder‐free system. Long‐termed, expanded SOX9+ RECs shared similar characteristics compared to those developed in the 3T3 feeder‐cell‐based system.^29^ This method supports the rapid acquisition of a large number of primitive cells and clinical applications for regenerative medicine with xeno‐free conditions.^59^

In recent decades, the mechanism of kidney regeneration in mammalians has been widely studied, and SOX9+ cells are found to be stimulated to participate in this process.^4^ The SOX9‐mediated mechanisms of repair are complex.^19^ Previous researchers published by our group^10^ and others^9^, ^17^ indicated that the proportion of SOX9+ progenitors was extensively increased after UIRI or partial nephrectomy, whose descendants contributed to multi‐segment nephron restoration, including proximal tubule, loop of Henle, distal tubule, collecting duct and the parietal layer of glomerulus. Similarly, our recent report showed that cultured human SOX9+ RECs could be in situ transplanted to generate functional proximal epithelium and early distal tubules.^29^ Altogether these works supported the pattern of SOX9+ RECs adopted to regenerate the renal epithelium by differentiation. In this article, we found SOX9+ RECs derived CM could replicate effective therapy of live cell transplantation, implying another pattern of renal restoration via secretory function.^5^

Moreover, here we clarified the potential molecular mechanism underlying the SOX9+ REC secretome‐mediated kidney repair by quantitative proteomic analysis. The experiments showed up‐regulation of several renoprotective factors, including S100A9. In a study by Mark C. Dessing et al., they observed that S100A9 deficiency led to sustained renal pathological state and dysfunction following renal UIRI,^58^ suggesting that S100A9 could lead to an advantageous phenotype in renal injury and repair in contrast to their role in cerebral injury and repair.^60^ To elucidate the benefits of S100A9 protein treatment, we performed the S100A9 protein administration in ischemic mice by I.V. injection. Our findings described that soluble S100A9 protein could replicate part of the regenerative effects of the full secretome, suggesting that a high level of S100A9 in the circulating environment contributes to renal homeostasis.^61^ Of note, the limitation of the study is that SOX9+ RECs secretome does not only contain soluble proteins, but also EVs. It would be sensible for future studies to include an EV administration study to investigate the therapeutic effects of renal damage. Moreover, previous studies reported many other types of adult stem/progenitor cells which contribute to kidney repair by cell replacement,^48^, ^62^, ^63^, ^64^, ^65^, ^66^, ^67^ it would be interesting to know whether they have a similar pro‐regeneration secretory function like SOX9+ RECs.

In conclusion, our study clarified the secretory function of SOX9+ RECs to regenerate renal epithelium, which elucidated a potential organ/tissue repair mechanism and provided novel translational opportunities for cell‐free strategies.

## AUTHOR CONTRIBUTIONS

Wei Zuo, Yu Ma and Yujia Wang designed the study. Hao Nie, Zixian Zhao, Dewei Zhou, Dandan Li, Xutao Liu and Yujia Wang involved in data collection and analysis. Wei Zuo, Hao Nie and Zixian Zhao drafted the manuscript. All authors read and approved the final manuscript.

## CONFLICT OF INTEREST

All authors declare that they have no conflict of interest.

## Supporting information


**Figure S1.** Cloning mice RECs from UUO injury model. (A) The percentage of SOX9+ cells at 0 and 1 dps. Data expressed as mean ± SD (*n* = 3 independent biological samples per group, **p < 0.01). (B) Representative serial images showed single cell‐derived td‐Tomato+ REC clones' expansion within 27 h. 1–3 indicated the clones with high proliferation capacity. Asterisks indicated the clones with low proliferation capacity. Scale bar, 500 μm. (C) REC colonies derived from sham and UUO injured kidneys stained with SOX9 and PAX2 antibodies. Scale bar, 50 μm.
**Figure S2.** Cell lineage analysis by single‐cell RNA‐sequencing in human subjects. (A) Twelve distinct cell clusters were visualized by UMAP plotting. (B) UMAP plot of cell clusters from different specimens of HC and AKI. The colour of the cells represented group origin. (C) Dot plots showed gene expression patterns of cluster‐enriched markers. (D) Enriched GO of SOX9+ RECs between HC and AKI subjects.
**Figure S3.** The signature of urinary SOX9+ cells in the published dataset. (A) Distribution and quantification of SOX9+ cell fractions. (B) Violin plots presented each cluster's marker genes and highlighted the selected marker genes for each cluster. (C) Heatmap exhibition of differentially secreted gene expressions between SOX9+ RECs and SOX9‐ cells.
**Figure S4.** Identifying human REC colonies from renal tissue and urine specimen. (A) Human REC colonies isolated from renal tissue (RECs‐tissue) of two patients with membranous nephropathy (MN) stained with indicated markers (representative images of *n* = 3 independent experiments). Scale bar, 20 μm. (B) Long‐term cultured RECs from urine stained with SOX9 and mature epithelial markers of ATP1A1 and CDH1 at early and late passage. Scale bar, 100 μm.
**Figure S5.** Quantitative analysis of tubular necrosis and glomerulus injury. (A) Quantitative scores of tubular necrosis post UIRI and RECs S.C. engraftment. (B) Quantitative scores of tubular necrosis post ADR and REC‐derived CM S.C. injection. (C) Quantification of the whole kidneys about FN1 and SYNPO after REC‐derived CM administration. Data shown in (A), (B) and (C) were represented as mean ± SD (*p < 0.05, **p < 0.01, ***p < 0.001, *n* = 3 independent biological samples per group).
**Figure S6.** SOX9+ RECs can repair the renal injury by I.P. injection. (A) Representative image of H&E‐staining after REC therapy. Sham, no surgery; UIRI, 3T3 cells pellets with 2–4^10^6^ after UIRI; UIRI+ RECs, equivalent‐number of REC pellets I.P. injection after UIRI. Scale bar, 100 μm. (B) Quantitative scores of tubular necrosis post UIRI and RECs I.P. engraftment. (C) The merged image of injured NOD‐SCID mouse kidney (left) and contralateral healthy kidney (right) 7 days after GFP‐RECs I.P. transplantation along the midabdominal line. (D,E) Immunostaining (D) and quantification (E) of the whole kidneys after REC I.P. treatment (*n* = 3 individual injury experiments using independent biological samples). Scale bar, 200 μm. (F) Serum creatinine level showed a reduction after REC treatment. Data shown in (B), (E) and (F) were represented as mean ± SD (*p < 0.05, ***p < 0.001, ****p < 0.0001, *n* = 3 independent biological samples per group, each group made in duplicate).
**Figure S7.** The CM of SOX9+ RECs reverse tubular epithelial cell damage caused by UIRI. (A) Representative H&E and IF post REC derived CM therapy like UIRI study. Sham, no surgery; UIRI, PBS subcutaneous injection after UIRI; UIRI + CM, equivalent volume CM subcutaneous injection after UIRI. Scale bar, 100 μm (HE); Scale bar, 200 μm (IF). (B) Quantitative scores of tubular necrosis post UIRI and RECs derived CM injection. (C) Quantification of representative markers after CM treatment. (D) Serum creatinine level showed a reduction after CM injection. (E) Isolation of RECs from patients followed by culture in SOX9+ RECs derived CM from two healthy volunteers (1# and 2#) resulted in higher cell viability by CCK‐8 test. Data shown in (B), (C), (D) and (E) were represented as mean ± SD (**p < 0.01, ***p < 0.001, ****p < 0.0001, *n* = 3 independent biological samples per group, each group made in duplicate).
**Figure S8.** GO analysis of secreted proteins in each cluster. (A–D) Dot plots of enriched GO terms in Cluster I (A), Cluster II (B), Cluster III (C) and Cluster IV (D). The X‐axis showed the fold enrichment of each GO term, whereas the colour denoted the *p*‐value and the size of the dot denoted the number of IDs assigned to each GO term.
**Figure S9.** Recombinant S100A9 protein restores kidney damage by I.V. injection. (A) H&E staining confirmed less kidney injury in the recombinant S100A9 protein treatment group. Scale bar, 50 μm. (B) Quantitative scores of tubular necrosis post UIRI and recombinant S100A9 protein therapy. (C) Quantification of the immunostained whole kidneys after recombinant S100A9 protein I.V. injection. (D) Serum creatinine level significantly decreased after recombinant S100A9 protein treatment. (E) Isolation of RECs followed by culture in DMEM or DMEM plus 10 μg/ml recombinant S100A9 protein. Cell viability validated by CCK‐8 test. Data shown in (B), (C), (D) and (E) were represented as mean ± SD (**p < 0.01, ***p < 0.001, ****p < 0.0001, *n* = 3 independent biological samples per group, each group made in duplicate).

## Data Availability

The data that support the findings of this study are available from the corresponding author upon reasonable request.
