# Supplementary material for: Activated SOX9+ renal epithelial cells promote kidney repair through secreting factors
Source: Cell Prolif. 2023 Jan 4;56(4):e13394. doi: 10.1111/cpr.13394 (PMC10068929; doi:10.1111/cpr.13394)
Supplement: Supplementary file 1 — Figure S1. Cloning mice RECs from UUO injury model. (A) The percentage of SOX9+ cells at 0 and 1 dps. Data expressed as mean ± SD (n = 3 independent biological samples per group, **p < 0.01). (B) Representative serial images showed single cell‐derived td‐Tomato+ REC clones' expansion within 27 h. 1–3 indicated the clones with high proliferation capacity. Asterisks indicated the clones with low proliferation capacity. Scale bar, 500 μm. (C) REC colonies derived from sham and UUO injured kidneys stained with SOX9 and PAX2 antibodies. Scale bar, 50 μm. Figure S2. Cell lineage analysis by single‐cell RNA‐sequencing in human subjects. (A) Twelve distinct cell clusters were visualized by UMAP plotting. (B) UMAP plot of cell clusters from different specimens of HC and AKI. The colour of the cells represented group origin. (C) Dot plots showed gene expression patterns of cluster‐enriched markers. (D) Enriched GO of SOX9+ RECs between HC and AKI subjects. Figure S3. The signature of urinary SOX9+ cells in the published dataset. (A) Distribution and quantification of SOX9+ cell fractions. (B) Violin plots presented each cluster's marker genes and highlighted the selected marker genes for each cluster. (C) Heatmap exhibition of differentially secreted gene expressions between SOX9+ RECs and SOX9‐ cells. Figure S4. Identifying human REC colonies from renal tissue and urine specimen. (A) Human REC colonies isolated from renal tissue (RECs‐tissue) of two patients with membranous nephropathy (MN) stained with indicated markers (representative images of n = 3 independent experiments). Scale bar, 20 μm. (B) Long‐term cultured RECs from urine stained with SOX9 and mature epithelial markers of ATP1A1 and CDH1 at early and late passage. Scale bar, 100 μm. Figure S5. Quantitative analysis of tubular necrosis and glomerulus injury. (A) Quantitative scores of tubular necrosis post UIRI and RECs S.C. engraftment. (B) Quantitative scores of tubular necrosis post ADR and REC‐derived CM S.C. [file CPR-56-e13394-s001.docx]

**Activated SOX9+ renal epithelial cells promote kidney repair through secreting factors**

Hao Nie, Zixian Zhao, Dewei Zhou, Dandan Li, Yujia Wang, Yu Ma, Xutao Liu,

Wei Zuo

**Supplementary Figure 1-9 and legend**

**
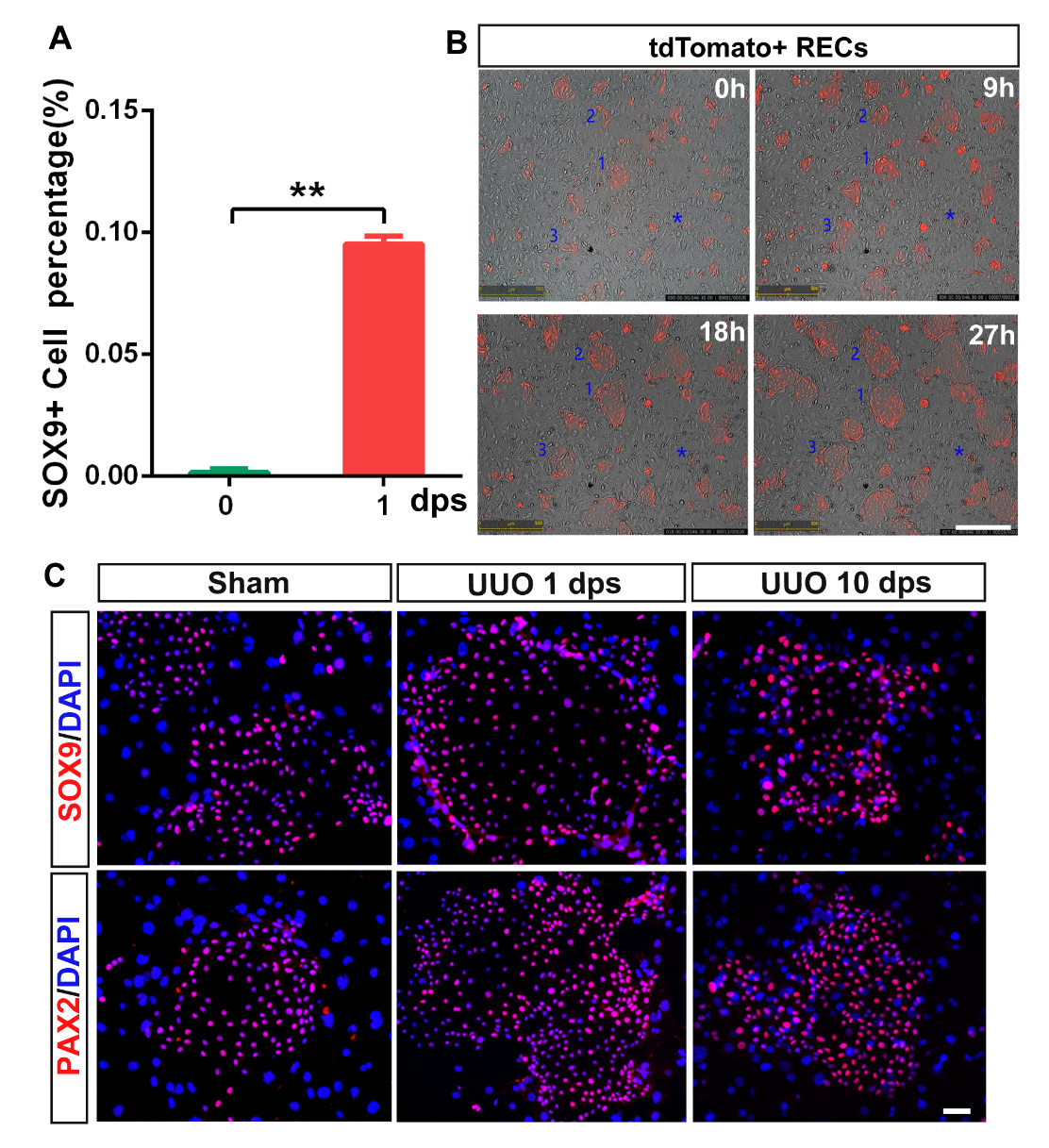
**

**Figure S1. Cloning mice RECs from UUO injury model.**

**A,** The percentage of SOX9+ cells at 0 and 1 dps. Data expressed as means ± SD (n=3 independent biological samples per group, ** P < 0.01). **B,** Representative serial images showed single cell-derived td-Tomato+ REC clones’ expansion within 27 hours. 1-3 indicated the clones with high proliferation capacity. Asterisks indicated the clone with low proliferation capacity. Scale bar, 500μm. **C,** REC colonies derived from sham and UUO injured kidneys stained with SOX9 and PAX2 antibodies. Scale bar, 50μm.

**
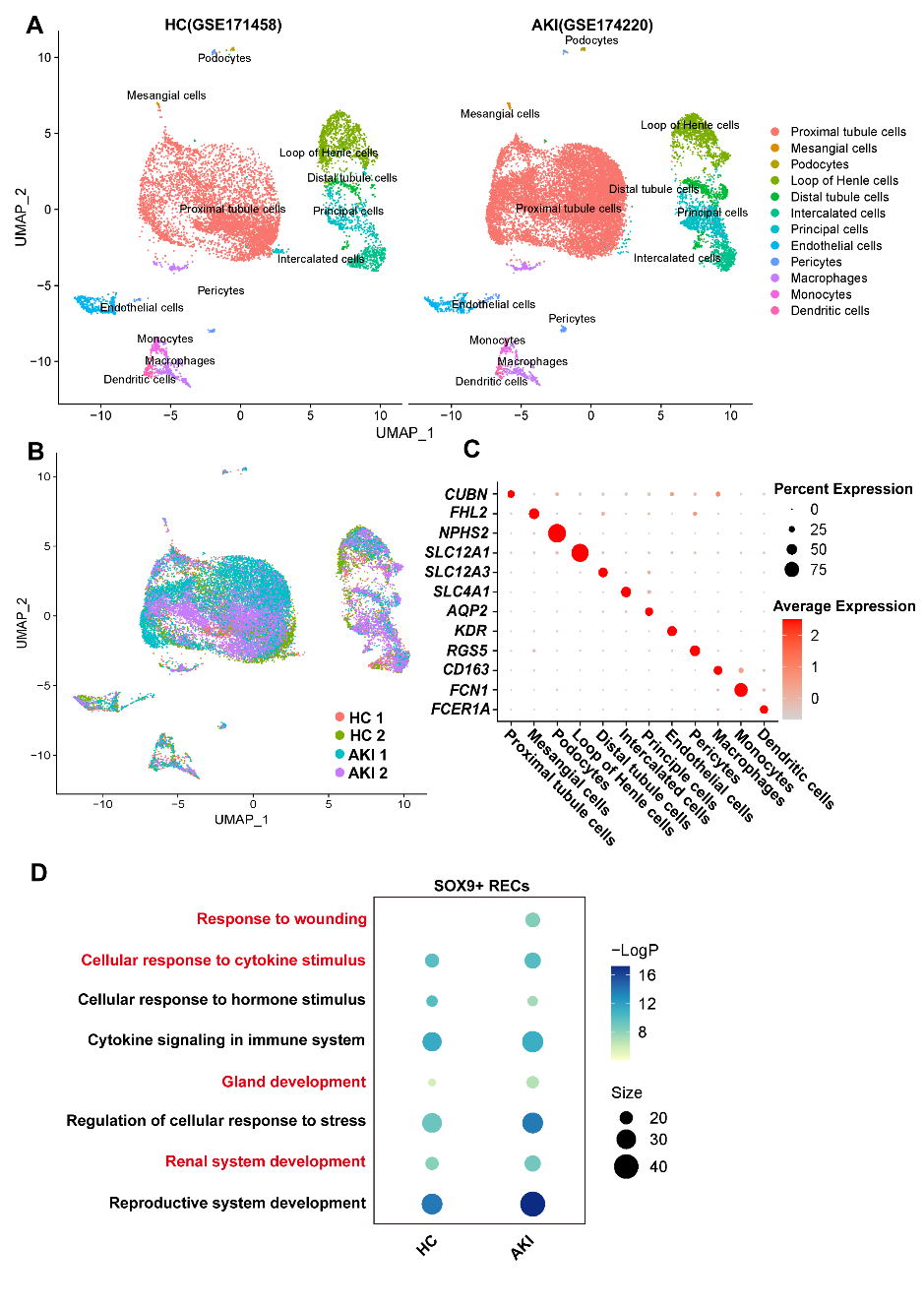
**

**Figure S2. Cell lineage analysis by single-cell RNA-sequencing in human subjects.**

**A,** Twelve distinct cell clusters were visualized by UMAP plotting. **B,** UMAP plot of cell clusters from different specimens of HC and AKI. The color of the cells represented group origin. **C,** Dot plots showed gene expression patterns of cluster-enriched markers. **D,** Enriched GO of SOX9+ RECs between HC and AKI subjects.

**
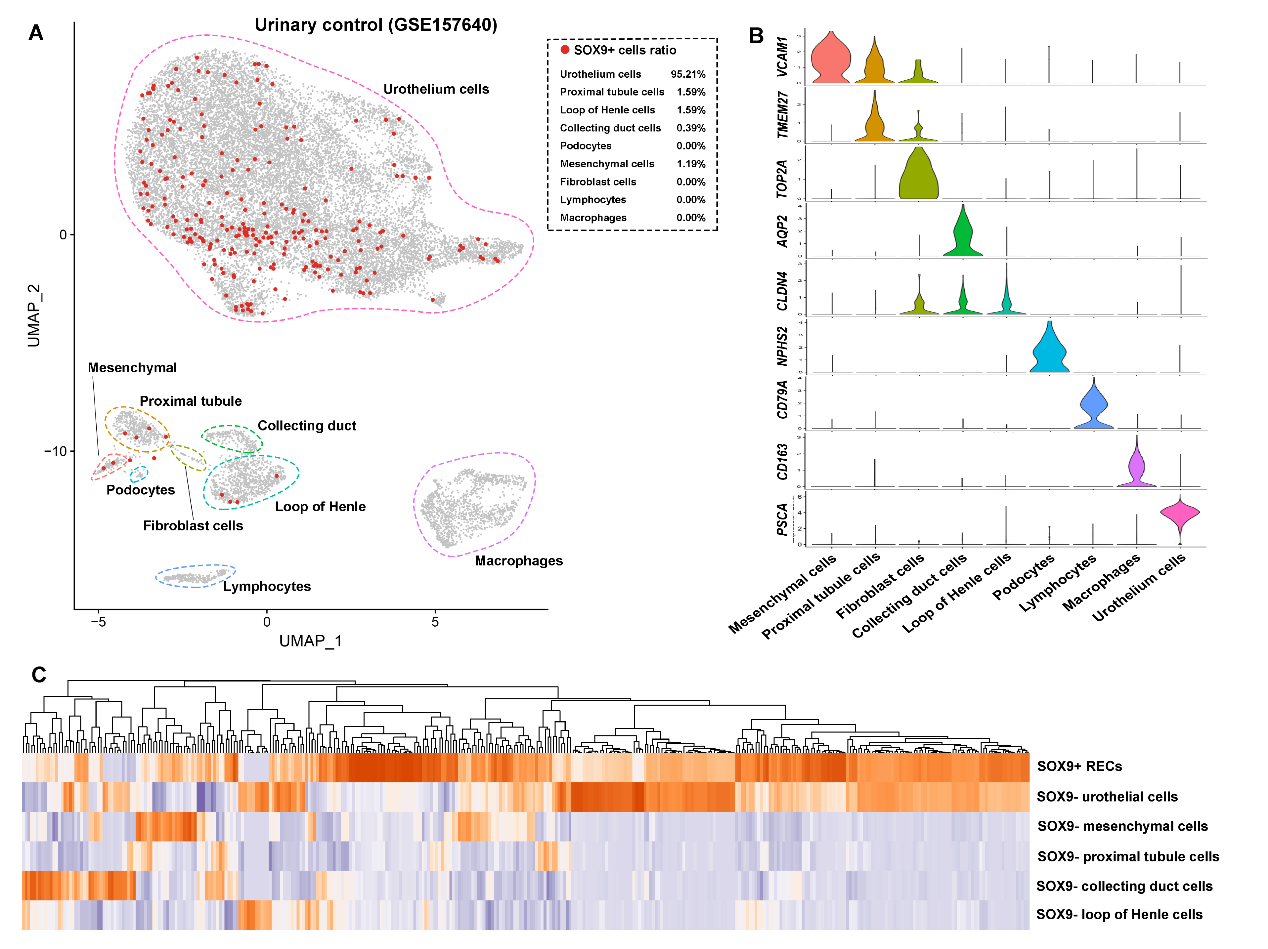
** **Figure S3. The signature of urinary SOX9+ cells in the published dataset.**

**A,** Distribution and quantification of SOX9+ cell fractions. **B,** Violin plots presented each cluster's marker genes and highlighted the selected marker genes for each cluster. **C,** Heatmap exhibition of differentially secreted gene expressions between SOX9+ RECs and SOX9- cells.

**
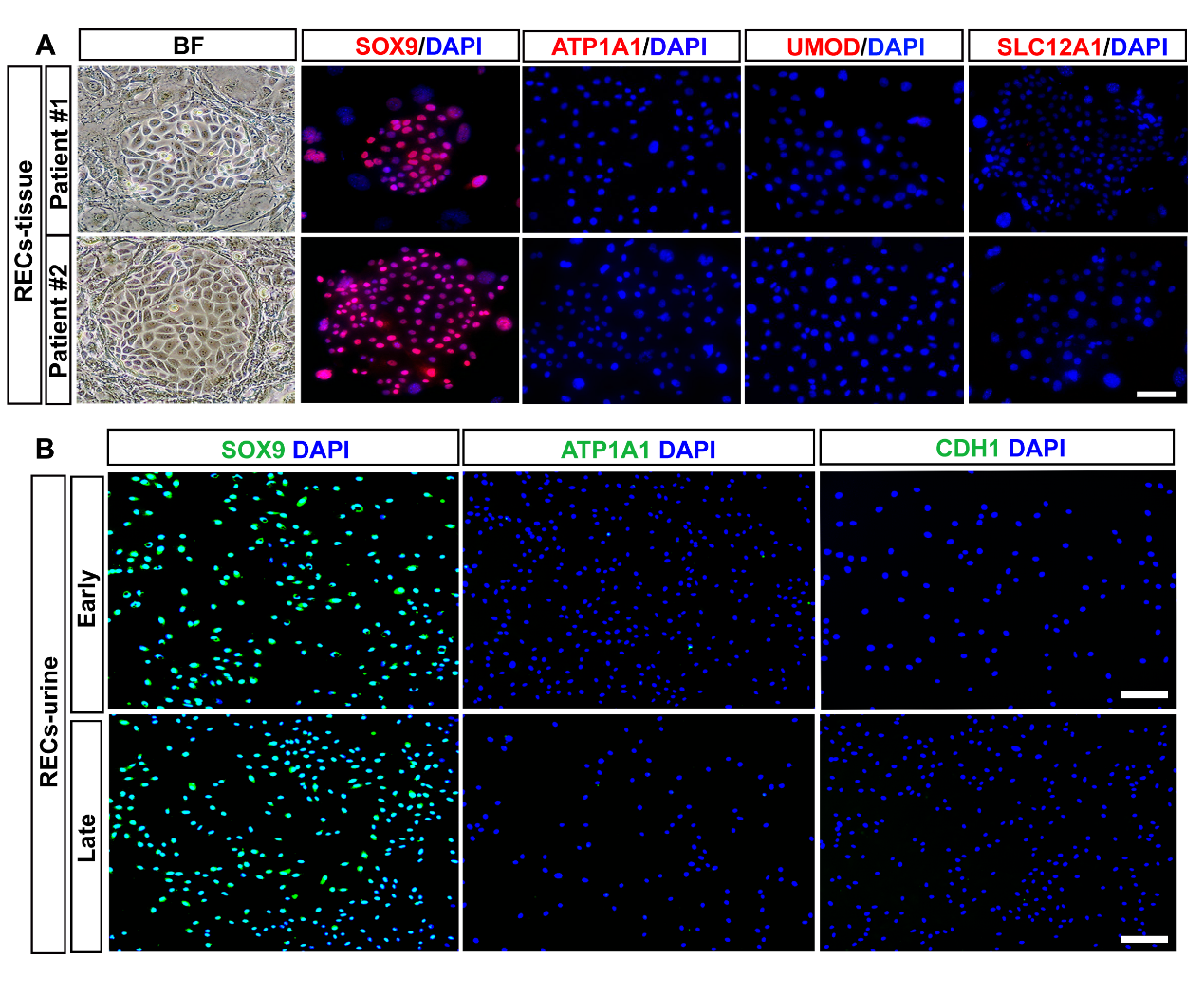
 Figure S4. Identifying human REC colonies from renal tissue and urine specimen.**

**A,** Human REC colonies isolated from renal tissue (RECs-tissue) of two patients with membranous nephropathy (MN) stained with indicated markers (representative images of n=3 independent experiments). Scale bar, 20μm. **B,** Long-term cultured RECs from urine stained with SOX9 and mature epithelial markers of ATP1A1 and CDH1 at early and late passage. Scale bar, 100μm.


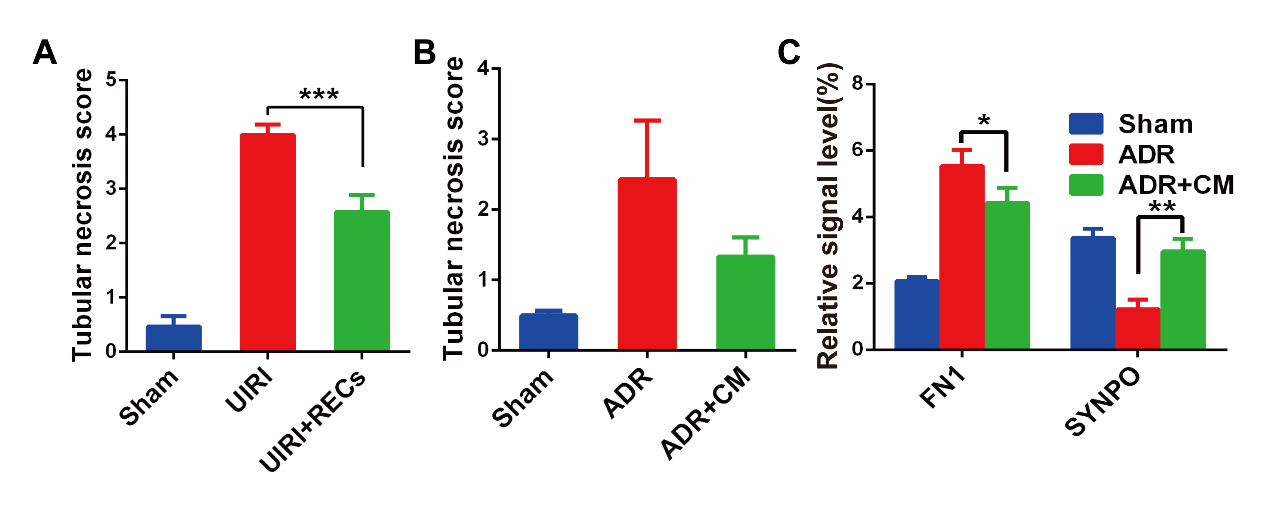


**Figure S5. Quantitative analysis of tubular necrosis and glomerulus injury.**

**A,** Quantitative scores of tubular necrosis post UIRI and RECs S.C. engraftment. **B,** Quantitative scores of tubular necrosis post ADR and REC-derived CM S.C. injection. **C,** Quantification of the whole kidneys about FN1 and SYNPO after REC-derived CM administration. Data shown in (A), (B) and (C) were represented as mean ± SD (*P < 0.05, ** P < 0.01, *** P < 0.001, n=3 independent biological samples per group).

**
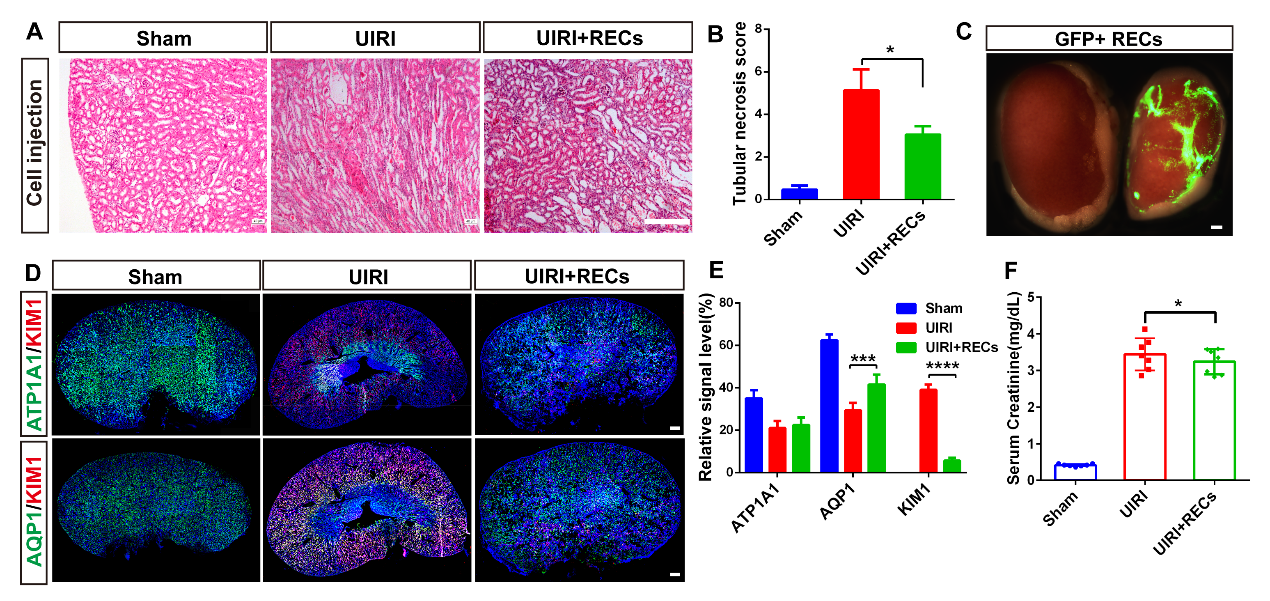
Figure S6. SOX9+ RECs can repair the renal injury by I.P. injection.**

**A,** Representative image of H&E-staining after REC therapy. Sham, no surgery; UIRI,3T3 cells pellets with 2-4^10^6^ after UIRI; UIRI+ RECs, equivalent-number of REC pellets I.P. injection after UIRI. Scale bar, 100μm. **B,** Quantitative scores of tubular necrosis post UIRI and RECs I.P. engraftment. **C,** The merged image of injured NOD-SCID mouse kidney(left) and contralateral healthy kidney (right) 7 days after GFP-RECs I.P. transplantation along the midabdominal line. **D, E** Immunostaining (D) and quantification (E) of the whole kidneys after REC I.P. treatment (n=3 individual injury experiments using independent biological samples). Scale bar, 200μm. **F,** Serum creatinine level showed a reduction after REC treatment. Data shown in (B), (E) and (F) were represented as mean ± SD (*P < 0.05, *** P < 0.001, **** P < 0.0001, n=3 independent biological samples per group, each group made in duplicate).

**
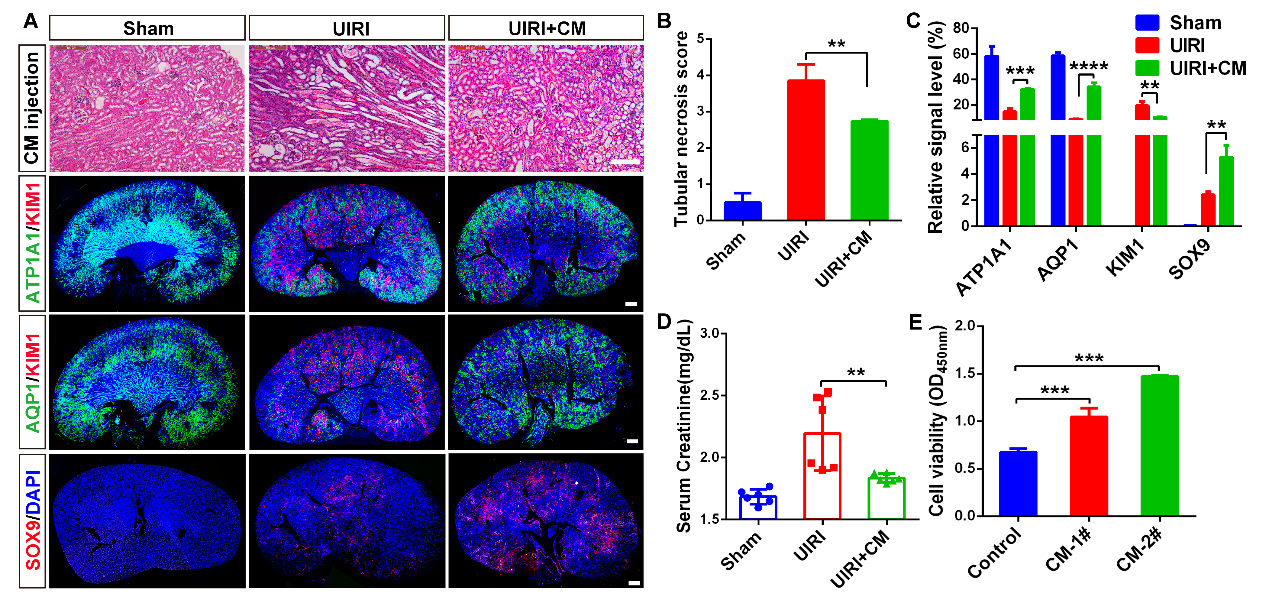
** **Figure S7. The CM of SOX9+ RECs reverse tubular epithelial cell damage caused by UIRI.**

**A,** Representative H&E and IF post REC derived CM therapy like UIRI study. Sham, no surgery; UIRI, PBS subcutaneous injection after UIRI; UIRI+CM, equivalent volume CM subcutaneous injection after UIRI. Scale bar, 100μm(HE); Scale bar, 200μm(IF). **B,** Quantitative scores of tubular necrosis post UIRI and RECs derived CM injection. **C,** Quantification of representative markers after CM treatment. **D,** Serum creatinine level showed a reduction after CM injection. **E,** Isolation of RECs from patients followed by culture in SOX9+ RECs derived CM from two healthy volunteers (1# and 2#) resulted in higher cell viability by CCK-8 test. Data shown in (B), (C), (D) and (E) were represented as mean ± SD (**P < 0.01, *** P < 0.001, **** P < 0.0001, n=3 independent biological samples per group, each group made in duplicate).

**
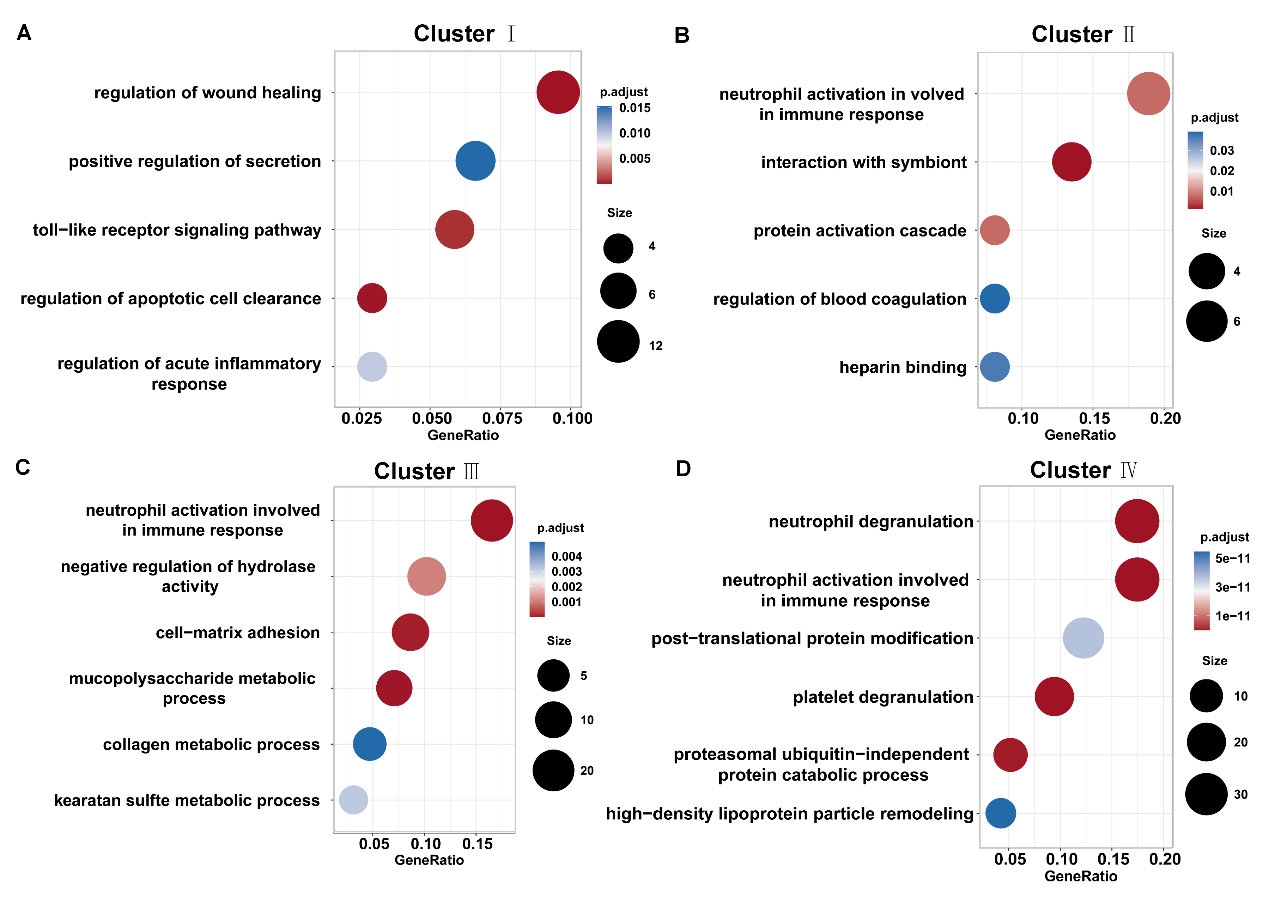
Figure S8. GO analysis of secreted proteins in each cluster.**

**A-D,** Dot plots of enriched GO terms in cluster Ⅰ (A), cluster Ⅱ (B), cluster Ⅲ (C), and cluster Ⅳ (D). The X-axis showed the fold enrichment of each GO term, whereas the color denoted the p-value and the size of the dot denoted the number of IDs assigned to each GO term.

**
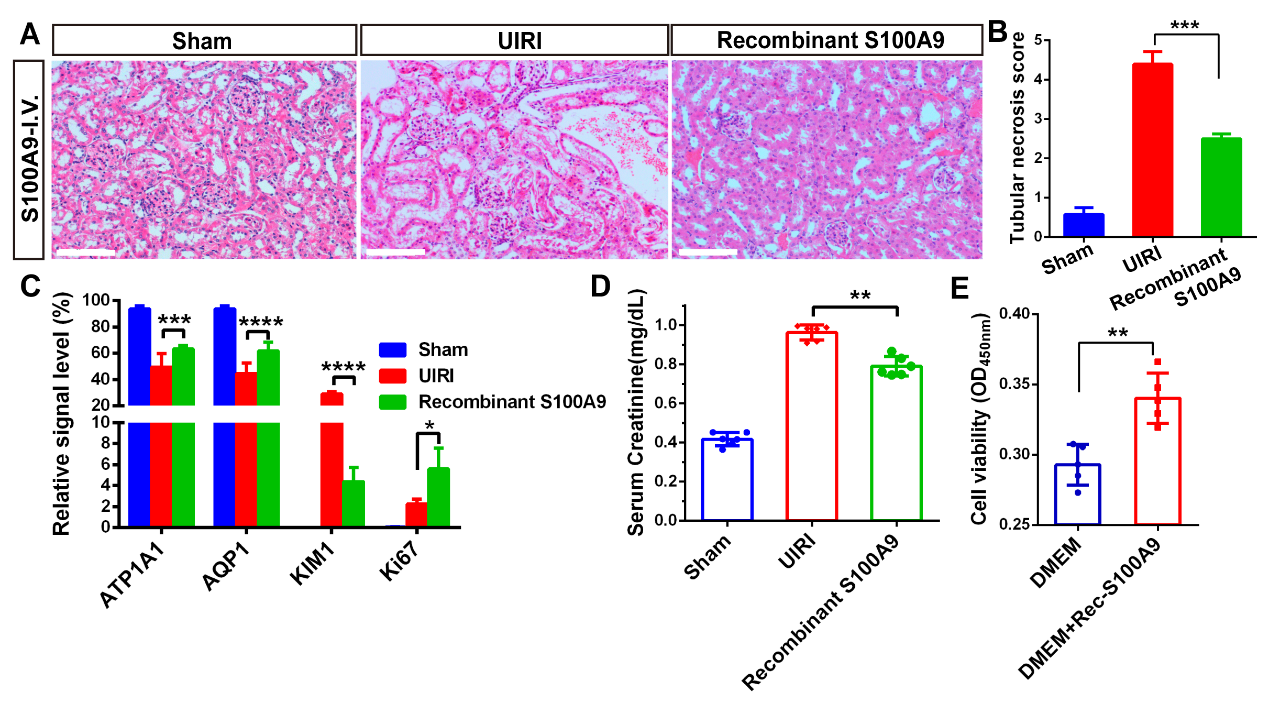
****Figure S9. Recombinant S100A9 protein restores kidney damage by I.V. injection.**

**A,** H&E staining confirmed less kidney injury in the recombinant S100A9 protein treatment group. Scale bar, 50μm. **B,** Quantitative scores of tubular necrosis post UIRI and recombinant S100A9 protein therapy. **C,** Quantification of the immunostained whole kidneys after recombinant S100A9 protein I.V. injection. **D,** Serum creatinine level significantly decreased after recombinant S100A9 protein treatment. **E,** Isolation of RECs followed by culture in DMEM or DMEM plus 10ug/ml recombinant S100A9 protein. Cell viability validated by CCK-8 test. Data shown in (B), (C), (D) and (E) were represented as mean ± SD (**P < 0.01, *** P < 0.001, **** P < 0.0001, n=3 independent biological samples per group, each group made in duplicate).
